# Impact of matrix-construction assumptions on quantitative overlap assessment in overviews: A meta-research study

**DOI:** 10.1017/rsm.2025.10056

**Published:** 2025-11-17

**Authors:** Javier Bracchiglione, Nicolás Meza, Dawid Pieper, Carole Lunny, Manuel Vargas-Peirano, Johanna Vicuña, Fernando Briceño, Roberto Garnham Parra, Ignacio Pérez Carrasco, Gerard Urrútia, Xavier Bonfill, Eva Madrid

**Affiliations:** 1 Department of Pediatrics, Obstetrics and Gynecology, Preventive Medicine and Public Health, Universitat Autònoma de Barcelona, Spain; 2 Iberoamerican Cochrane Centre, Institut de Recerca Sant Pau (IR SANT PAU), CIBERESP, Spain; 3 Interdisciplinary Centre for Health Studies (CIESAL), Universidad de Valparaíso, Chile; 4 Cochrane Evidence Synthesis Unit Iberoamerica, Iberoamerican Cochrane Centre, Spain; 5 Institute for Health Services and Health System Research (IVGF), Faculty of Health Sciences Brandenburg (FGW), Brandenburg Medical School Theodor Fontane, Germany; 6 Center for Health Services Research (ZVF-BB), Brandenburg Medical School Theodor Fontane, Germany; 7 Knowledge Translation Program, St Michaels Hospital, Unity Health Toronto, The University of British Columbia, Canada; 8 Precision for Medicine, Canada; 9 Institut de Recerca Sant Pau (IR SANT PAU), Hospital de la Santa Creu i Sant Pau, Spain; 10 School of Medicine, Universidad de Valparaiso, Chile

**Keywords:** corrected covered area, matrix of evidence, overlap, overviews of systematic reviews, systematic reviews as topic

## Abstract

Overlap of primary studies among multiple systematic reviews (SRs) is a major challenge when conducting overviews. The corrected covered area (CCA) is a metric computed from a matrix of evidence that quantifies overlap. Therefore, the assumptions used to generate the matrix may significantly affect the CCA. We aim to explore how these varying assumptions influence CCA calculations. We searched two databases for intervention-focused overviews published during 2023. Two reviewers conducted study selection and data extraction. We extracted overview characteristics and methods to handle overlap. For seven sampled overviews, we calculated overall and pairwise CCA across 16 scenarios, representing four matrix-construction assumptions. Of 193 included overviews, only 23 (11.9%) adhered to an overview-specific reporting guideline (e.g. PRIOR). Eighty-five (44.0%) did not address overlap; 14 (7.3%) only mentioned it in the discussion; and 94 (48.7%) incorporated it into methods or results (38 using CCA). Among the seven sampled overviews, CCA values varied depending on matrix-construction assumptions, ranging from 1.2% to 13.5% with the overall method and 0.0% to 15.7% with the pairwise method. CCA values may vary depending on the assumptions made during matrix construction, including scope, treatment of structural missingness, and handling of publication threads. This variability calls into question the uncritical use of current CCA thresholds and underscores the need for overview authors to report both overall and pairwise CCA calculations. Our preliminary guidance for transparently reporting matrix-construction assumptions may improve the accuracy and reproducibility of CCA assessments.

## Highlights

**What is already known?**

- Overlap of primary studies is a major methodological challenge when conducting overviews of systematic reviews (SRs).
- The corrected covered area (CCA) quantifies the percentage of overlap based on a matrix of evidence—a structured table that lists primary studies and indicates which ones are included in each SR.
- This measure is useful for understanding whether multiple SRs are providing largely the same information or if they offer unique contributions to the overall body of evidence.

**What is new?**

- We are the first to empirically demonstrate how key methodological choices—such as scope definitions, handling of structural missingness, and publication‐thread adjustments—can alter CCA values across multiple overview scenarios.
- Drawing on our scenario analyses, we propose the field’s first transparent protocol for constructing evidence matrices and reporting both overall and pairwise CCA, thereby enabling reproducible overlap assessments.
- By highlighting the unexplored methodological basis of existing CCA cut-offs (<5%, 5–10%, 10–15%, ≥15%), we provide the initial empirical foundation for refining these thresholds and guiding more robust methodological decisions in overviews.

**Potential impact for RSM readers**

- We demonstrate for the first time how critical decisions in evidence‐matrix construction (scope, structural missingness, publication threads) quantitatively alter CCA values, encouraging methodologists to scrutinise and document these choices.
- We provide actionable advice for handling multiple publications (‘publication threads’) and structural missingness, strengthening the rigor and trustworthiness of overlap assessments in overviews.
- We highlight the complementary value of reporting both overall and pairwise CCA metrics to capture the full overlap phenomenon, preventing misleading interpretations driven by aggregate measures alone.
- We challenge the arbitrary nature of existing CCA cut‐offs (<5% slight, 5–10% moderate, 10–15% high, ≥15% very high) and lay the groundwork for methodological validation.
- We introduce a clear protocol for transparently reporting matrix construction by expliciting methodological assumptions for both overall and pairwise CCA calculations, enhancing reproducibility and comparability across overview studies.

## 1 Introduction

Overviews are a form of evidence synthesis that uses systematic reviews (SRs) as their unit of search, inclusion and analysis.^1^ Because each SR is based on a set of primary studies, an overview that includes multiple SRs on the same research question is likely to have identical or partially overlapping primary studies. This redundancy, known as ‘overlap of primary studies’, poses a significant challenge when conducting overviews.^1^^–^ ^8^ Primary study overlap may bias the overview findings by disproportionately amplifying the influence of specific studies. Moreover, overlap leads to artificially precise estimates due to the repeated inclusion of identical effect estimates derived from the same primary studies.^1^ When multiple SRs include the same primary studies, each duplicated study contributes its effect estimate more than once to the overall synthesis. Because meta-analytic methods weight studies by the inverse of their variance, these repeated entries artificially inflate the apparent sample size and reduce the estimated variance. As a result, confidence intervals around the pooled effect become narrower—not because the evidence is actually stronger or more consistent, but simply because identical data points have been counted multiple times. This misrepresents the degree of precision, increasing the risk of overconfident conclusions and potentially misleading decision-makers about the true estimates of effects.

There is no clear consensus on how to manage primary study overlap (hereafter referred to as overlap). Several authors do not consider overlap when conducting overviews or simply acknowledge it as a potential limitation in their discussion.^2^^,^ ^5^^,^ ^9^^–^ ^12^ By failing to formally assess and adjust for overlap, their syntheses remain vulnerable to bias and can produce misleading conclusions. Others propose addressing overlap by making eligibility or analytical decisions based on the presence of potential overlap—for instance, analysing only one SR (e.g. the most recent, the most comprehensive, or the highest quality) when multiple SRs share a common primary study.^3^^,^ ^4^ However, this approach risks losing valuable information provided by the primary studies included in the reviews that are excluded or not analysed. Moreover, there is no universally accepted guidance for choosing among multiple SRs, and the most appropriate choice often depends on the body of evidence of the specific clinical specialty or the particular public health concern being addressed.^13^^,^ ^14^

Some authors have proposed meta-analysing data directly from the primary studies, instead of pooled data from the SRs.^12^ With this approach, the potential for bias or distorted imprecision derived from overlap is solved, but it raises the question of why conducting an overview in the first place, and not a SR directly. Beyond simply providing a descriptive or statistical summary of the primary studies included in SRs, a critical role of overviews is to comprehensively display, analyse, and discuss the available evidence syntheses, in an effort to facilitate comparisons across them by explaining their possible differences and similarities, as well as strengths and limitations. Beyond these challenges, overviews also offer important advantages. They provide a comprehensive map of existing evidence syntheses, making it possible to identify where reviews converge or diverge in their conclusions, reveal methodological differences that may explain discordant findings, and highlight both duplication and research gaps. The unique contribution of overviews lies in critically analysing and comparing the body of SRs, rather than statistically combining their results, which may be particularly valuable for decision-makers and guideline developers. Therefore, assessing overlap is essential—not only to decide whether to pool primary study data, but to ensure that the interpretive comparisons provided by overviews reflect the actual evidence base rather than artefacts of duplication.

Another approach to address overlap is to include all the available SRs and explicitly describe its magnitude. Visual representations can be used, such as Venn or Euler diagrams, node-link graphs, bar plots, and upset plots.^15^ Among overlap‐mapping methods, the evidence matrix is most commonly used.^1^ An evidence matrix (or citation matrix) is a simple two-dimensional table: one axis lists the SRs and the other lists the primary studies, and each cell is filled in to show when a given study appears in a particular SR.^2^ While this clear visualisation works well for small numbers of reviews, it rapidly becomes unwieldy and hard to interpret as more SRs and studies are added.

The corrected covered area (CCA) is a quantitative approach to describe overlap, based on a matrix of evidence.^2^^,^ ^16^ CCA is a quantitative measure of how much primary‐study overlap exists across a set of SRs, expressed as a percentage. It is calculated by comparing the total number of ‘occurrences’ of primary studies (i.e., the sum of all studies counted in every SR) with the number of unique primary studies and the number of SRs included. Conceptually, CCA answers the question: ‘Of all the study entries in my overview, what proportion are duplicates?’ A higher CCA indicates more repeated studies and therefore greater overlap.

To interpret CCA, researchers have adopted the following (albeit arbitrary) thresholds^2^:

- <5%: slight overlap
- 5–10%: moderate overlap
- 10–15%: high overlap
- >15%: very high overlap

These cut-offs have been used by some authors as practical guidance when planning or conducting overviews, helping teams decide whether overlap is trivial or whether it must be formally addressed to avoid bias.

Because these percentage thresholds were chosen arbitrarily and have not been subjected to meta-epidemiological validation, we still lack empirical evidence on how varying degrees of overlap bias overview results. Standardising the matrix assumptions for CCA calculations may guide researchers on how to interpret overlap in quantitative synthesis—whether using predefined thresholds or considering CCA as a broad indicator of overlap—helping to determine whether an overview could definitively answer a clinical question.

Therefore, it is essential to establish a clear method for building an evidence matrix before measuring overlap with the CCA. Constructing an evidence matrix involves several methodological assumptions, including how rows and columns are defined (e.g. whether each row represents an individual reference or the set of references related to a primary study), how CCA calculations are performed (overall or pairwise CCA), and how the unit of analysis is determined (overview-level or outcome-level).^17^ So far, no study has assessed the impact of these assumptions. Our research aims to examine how varying assumptions in constructing matrices of evidence influence CCA calculations when assessing primary study overlap in overviews.

## 2 Methods

We conducted a methodological review, reporting our publication based on relevant items in the ‘Preferred Reporting Items for Systematic reviews and Meta-Analyses’ (PRISMA) guidelines.^18^^,^ ^19^ The protocol for this study was prospectively registered.^17^

### 2.1 Eligibility criteria

We included overviews, defined as any type of evidence synthesis in which the unit of searching, inclusion, and analysis is SRs.^1^ We only considered overviews focused on health interventions of any type published in peer-reviewed journals that explicitly listed the references of the included SRs. We excluded other types of evidence syntheses (such as individual SRs and broad literature reviews including both SRs and primary studies), as well as overviews with a non-intervention focus (e.g. diagnostic, prognosis).

### 2.2 Search methods and selection of studies

We searched MEDLINE via PubMed and the Cochrane Database of Systematic Reviews using previously validated filters for retrieving overviews (Appendix 1).^20^ We limited our search to articles published between January 1, 2023, and April 1, 2023. We set the lower chronological limit considering that the latest version of the ‘Cochrane Handbook for Systematic Reviews of Interventions’, which introduced guidance for conducting overviews, was first published in 2019, and the ‘Preferred Reporting Items for Overviews of Reviews’ (PRIOR) statement was released in 2022.^1^^,^ ^21^

Two authors (among JB, NM, MV, JV, RGP, and IPC) conducted the selection process, determining eligibility first by title and abstract, and then by full-text assessment. Discrepancies were solved through discussion and consensus.

### 2.3 Data extraction and analysis

Data extraction was conducted in two steps. First, one author (JB) extracted the study characteristics for all the identified overviews, and a second author (MV, JV) cross-checked them. The variables included:

- Year of publication.
- Reporting guideline used, classified as overview-specific (i.e. those stating overlap of primary studies within their reporting statements, such as PRIOR) or non-overview specific (i.e. those developed for other types of evidence syntheses, such as PRISMA).
- Main outcome.
- Number of included SRs.
- Methods to address overlap, according to the ‘Methods for Overviews of Reviews’ (MOoR) framework (further detailed in Appendix 2), which may be done:
  - In the ‘eligibility criteria’ step (by including reviews are selected based on pre-specified criteria, excluding those without unique primary studies, or including all the reviews).
  - In the ‘data extraction’ step (by extracting data from all reviews, or only from selected ones according to prespecified criteria).
  - In the ‘risk of bias assessment’ step (by selecting reviews according to their risk of bias).
  - In the ‘synthesis and presentation and summary of findings’ step (by selecting reviews according to prespecified criteria, or using methods to visualise or quantify overlap).
- Scope classification is defined as either:^22^
  - Narrow: covering a single population and a single intervention or comparison (e.g. ‘*Clinical Efficacy of Platelet Derivatives in Periodontal Tissue Regeneration’*).^23^
  - Broad: covering multiple populations or multiple interventions (e.g. ‘*Non-pharmacological interventions for self-management of fatigue in adults*’).^24^
  - Borderline cases, such as overviews including multiple drugs within a single treatment class (e.g. ‘Chemotherapy and targeted therapy for advanced biliary tract cancers’), were classified as narrow if the interventions were conceptually similar and addressed a single clinical question (see Appendix 3 for the full list of overviews and their classifications)^25^

We summarised these data descriptively in tables. As a post hoc analysis, we compared the association between using a reporting guideline tailored for overview methodology (i.e. a guideline mentioning overlap specifically in their reporting criteria, such as PRIOR^21^ or PRIO-harms^26^) and whether the authors considered overlap within the identified overviews.

After summarising these data, we selected a purposive sample of overviews to create matrices of evidence. For this, we considered overviews that had different scopes (broad and narrow), and with a heterogeneous number of included SRs. The sample did not consider overviews that solved the overlap during the eligibility criteria step (i.e. excluded SRs based on overlap), as overlap is not expected to appear in those cases. One author (JB, FB) created the matrices of evidence for each overview, and a second author (NM) cross-checked for accuracy. The initial matrix considered all the SRs included in the overview, as well as all the references of the primary studies within those SRs.

For each overview, we tested different assumptions that authors may have considered when constructing matrices of evidence.^2^^,^ ^16^^,^ ^27^^–^ ^40^ These assumptions include factors related to the level of analysis (overview-level versus outcome-level) and how the matrix was constructed (adjustments for scope, publication threads, and structural missingness), as defined in Table 1. By considering all possible combinations of these assumptions, we created a total of 16 matrices per overview. These scenarios are detailed in Table 2.

**Table 1:** Important overlap definitions

Concept		Definition	Further considerations
General concepts	Matrix of evidence^2^	A grid that maps the SRs against their respective primary studies.	A matrix of evidence (also called a citation matrix) is a two-dimensional table used to quantify overlap among systematic reviews (SRs) when computing the Corrected Covered Area (CCA). In this matrix, rows represent individual SRs included in an overview, columns represent all unique primary studies cited across those SRs, and cells contain a mark (often ‘1’ for presence, ‘0’ for absence) indicating whether a given primary study appears in a particular SR.
	Corrected covered area (CCA)^2^	A metric that quantifies the degree of overlap of primary studies across different SRs, expressed as a percentage.	CCA is calculated using data from a matrix of evidence, as follows: 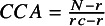 Where: 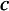 : the total number of included systematic reviews (columns). 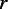 : the total number of unique index publications (rows). 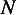 : the total number of primary studies (including duplicates of the same primary study across reviews).A higher CCA represents a higher degree of overlap.
Unit of analysis for the overlap assessment	Overview-level overlap assessment^2^	The construction of one matrix for the whole overview, considering all the included SRs.	This approach treats the entire overview as the unit of analysis.The CCA is derived from one matrix for the whole overview.
	Outcome-level overlap assessment^36^^–^ ^39^	The construction of one matrix per outcome, considering only the SRs providing data for the specific outcome.	This approach treats each outcome within the overview as the unit of analysis.A CCA is derived from multiple matrices (one matrix for each outcome).
Assumptions for creating a matrix of evidence and calculating CCA	Scope adjustment^40^	The consideration—and inclusion in the matrix—only of the primary studies that provide useful data for a given outcome within an overview.	Some SRs within an overview may contain primary studies that do not provide valuable data for the overview. We will consider a matrix as non-adjusted by scope if it includes all the primary studies within the SRs, irrespective of whether they provide useful data for the overview. We will consider a matrix as adjusted by scope if it includes only the primary studies providing data for at least one outcome of interest.
	Publication-thread adjustment^27^^–^ ^35^	The consideration of each set of references related to a single primary study as a unique row in a matrix of evidence, instead of multiple rows.	When a single research project gives rise to several journal articles—such as an initial protocol paper, one report on outcome A, another on outcome B, and perhaps a long-term follow-up—this is known as multiple publications (or sometimes ‘salami slicing’). Although each paper appears distinct, they all stem from the same underlying study and patient cohort. As such, data from the same primary study may be split and published in multiple publications. We will consider a matrix as non-adjusted by publication-thread (‘studification’) if each row represents a unique reference (in which case the same study could be represented by multiple rows). We will consider a matrix as adjusted by publication-thread if each row of the matrix represents a set of references related to the same primary study.
	Structural missingness adjustment^16^^,^ ^37^^,^ ^38^	The exclusion of primary studies that cannot be included in the SRs due to methodological constraints for purposes of CCA calculation.	Some intersections in a matrix of evidence may be blank for valid reasons, which is referred to as ‘structural missingness’.For this study, publication timing will be considered as the only valid and unequivocal methodological constraint (e.g., a primary study published in 2024 cannot be included in an SR published in 2021).In practical terms, structural missingness adjusts the original CCA formula by subtracting the number of intersections considered as structurally missed from the denominator. This modified CCA formula is: 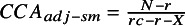 Where 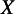 is the number of intersections considered as structural missingness. We will consider a CCA calculation as non-adjusted by structural missingness if it uses the original CCA formula. We will consider a CCA calculation as adjusted by structural missingness if it uses this modified CCA formula.
CCA calculation approach	Overall CCA calculation^2^	The calculation of a unique CCA value for a given matrix of evidence.	The overall CCA calculation considers every SR and primary study within the matrix of evidence.
	Pairwise CCA calculation^11^^,^ ^16^	The calculation of multiple CCA values for a given matrix of evidence, each representing the specific overlap for every possible pair of SRs.	The pairwise CCA calculation considers the theoretical construction of a matrix of evidence for each possible pair of SRs. Each of those matrices contains only the primary studies relevant to that specific pair of reviews.Since this approach provides multiple CCA values, the results are summarised using box plots, displaying the medians and interquartile ranges for the calculated CCAs across different pairs of SRs.

CCA: corrected covered area; SR: systematic review.

**Table 2:** Different scenarios planned for overlap analysis through CCA

Scenario number^a^	Level of analysis	Scope adjustment^b^	Publication thread adjustment	Structural missingness adjustment
1	Overview	✗	✗	✗
2	Overview	✓	✗	✗
3	Overview	✗	✓	✗
4	Overview	✗	✗	✓
5	Overview	✓	✓	✗
6	Overview	✓	✗	✓
7	Overview	✗	✓	✓
8	Overview	✓	✓	✓
9	Outcome	✗	✗	✗
10	Outcome	✓	✗	✗
11	Outcome	✗	✓	✗
12	Outcome	✗	✗	✓
13	Outcome	✓	✓	✗
14	Outcome	✓	✗	✓
15	Outcome	✗	✓	✓
16	Outcome	✓	✓	✓

*Note*: ✓: adjusted; ✗: non-adjusted; CCA: corrected covered area.

a We conducted overall and pairwise CCA calculations for each scenario.

b Scope adjustment considers a matrix including only the reviews relevant to the main outcome of the review.

Once we constructed a matrix for each scenario, we calculated the overall and pairwise CCA using the ‘Graphical Representation of Overlap for OVErviews’ (GROOVE) tool.^16^ We present the results of both assessments in boxplots for each scenario and overview. We calculated the difference between the maximum and minimum CCA values across the different scenarios to estimate the potential impact of different assumptions on the CCA value.

Because existing CCA cut-offs are arbitrary, we did not intend to adopt strict thresholds to determine the importance of change. Our narrative labels describe the magnitude of variation across scenarios (e.g. as ‘minimal’, ‘moderate’ or ‘marked’) based on the absolute observed change in CCA for that overview, with the intention of providing readers with contextual information. To improve transparency and repeatability, we explicitly report the exact change in CCA values for every overview, in every scenario. With the intent of improving clarity in the text, we have used the following operational (non-normative) categories for the absolute change in CCA across scenarios: minimal, if the change in CCA is equal or below 5%; moderate, if above 5% and below 10%; and marked if equal or above 10%. These categories are reported only as descriptive aids and do not imply formal benchmarks.

## 3 Results

### 3.1 Search results

**Figure 1:**
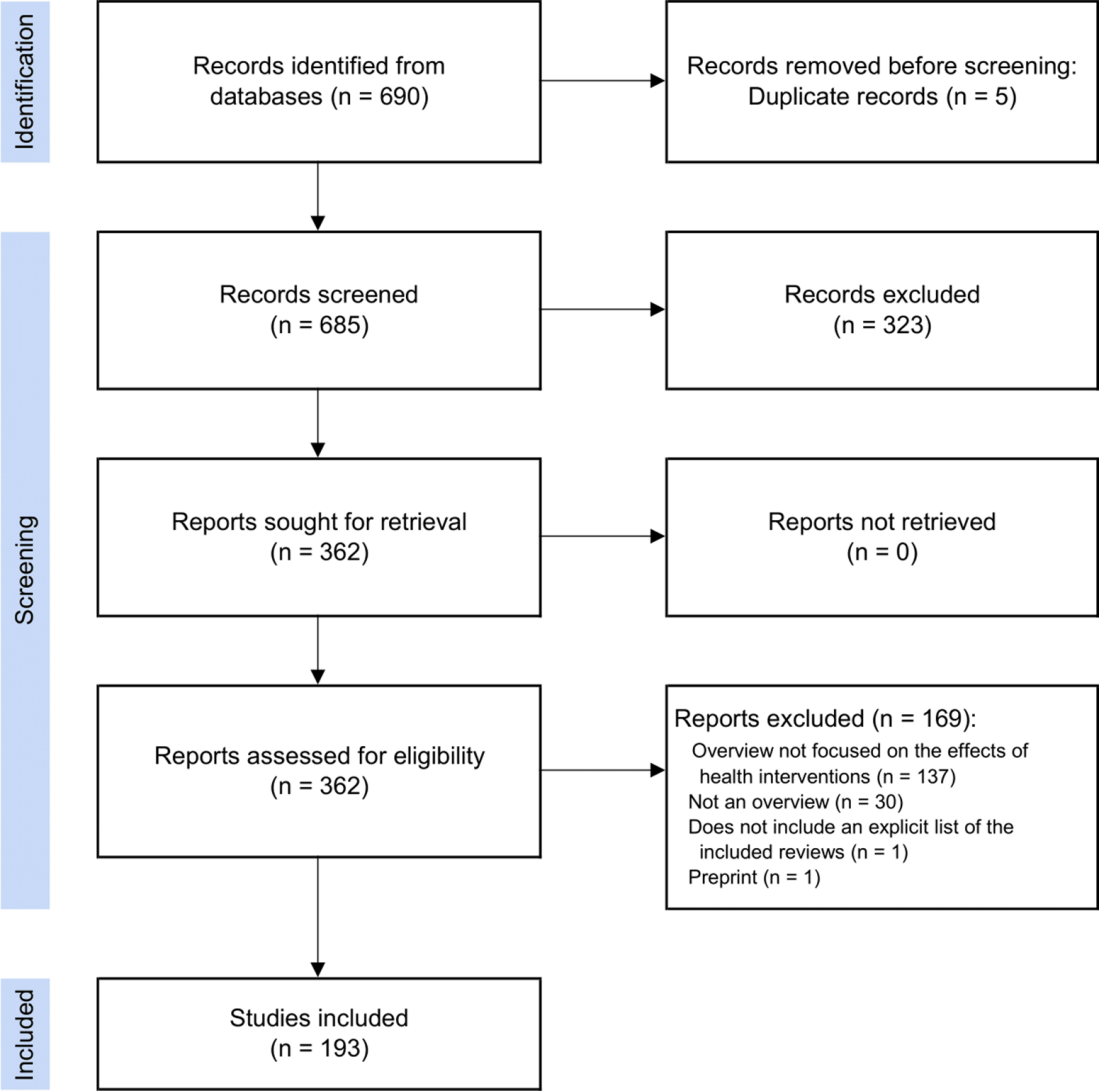
PRISMA flow chart summarising the selection process.

Our search strategy yielded a total of 690 references. After removing duplicates, we screened 685 records by title and abstract, excluding 323. A total of 362 reports were retrieved, of which we excluded 169 due to the following reasons: non-intervention focus (n = 137), not being an overview (n = 30), no explicit list of included reviews (n = 1), and preprint not published in a peer-reviewed journal (n = 1). Appendix 3 provides the list of excluded studies, with reasons. Ultimately, 193 studies met our eligibility criteria. Figure 1 summarises the selection process.

### 3.2 Description of the identified overviews

The 193 identified overviews included a median of 15 SRs (IQR: 10–27, range 0–101), with most classified as having a broad scope (n = 129, 66.8%). Most overviews (n = 117, 60.6%) used a reporting guideline not specific to overviews (e.g. PRISMA), with only 23 (11.9%) using an overview-specific reporting guideline (e.g. PRIOR). A significant proportion (n = 85, 44.0%) did not mention overlap in their manuscript, while 14 (7.3%) addressed it only in the discussion section. A total of 94 (48.7%) overviews incorporated overlap within their methods or results sections, mainly in the ‘Synthesis and presentation and summary of findings’ step (n = 61, 31.6%). There was no statistically significant association between the guideline used and the consideration of overlap (p = 0.104). The main characteristics of the identified overviews are summarised in Table 3 and detailed in Appendix 4.

**Table 3:** Summary of the main characteristics of the included overviews

Characteristics	Total overviews (n = 193)
Median included SRs (IQR)	15 (10–27)
**Scope of the population**	
Narrow	138 (71.5%)
Broad	55 (28.5%)
**Scope of the interventions or comparisons**	
Narrow	93 (48.2%)
Broad	100 (51.8%)
**Overall scope** ^**a**^	
Narrow	64 (33.2%)
Broad	129 (66.8%)
**Reporting guideline used**	
Not reported	53 (27.5%)
SR-specific	117 (60.6%)
PRISMA	114 (59.1%)
PRISMA-ScR	2 (1.0%)
MOOSE	1 (0.5%)
Overview-specific	23 (11.9%)
PRIOR	17 (8.8%)
PRIO-harms	6 (3.1%)
**Overlap mention**	
No mention of overlap	85 (44.0%)
Methods or results section	94 (48.7%)
Discussion section	14 (7.3%)
**Specific steps in which overlap was addressed** ^**b**^	
‘Eligibility criteria’ step	10 (5.2%)
‘Data extraction’ step	2 (1.0%)
‘Assessment of risk of bias’ step	0 (0.0%)
‘Synthesis and presentation and summary of findings’ step	61 (31.6%)
More than one step	19 (9.8%)
Not specified	2 (1.0%)

IQR: Interquartile range; MOOSE: ‘Meta-analysis Of Observational Studies in Epidemiology’; PRIO-harms: ‘Preferred Reporting Items for Overviews of systematic reviews including harms’; PRIOR: ‘Preferred Reporting Items for Overviews of Reviews’; PRISMA: ‘Preferred Reporting Items for Systematic reviews and Meta-Analyses’; PRISMA-ScR: ‘PRISMA Extension for Scoping Reviews’; SR: Systematic review.

a Overall scope was considered ‘narrow’ if both the population and the intervention were narrow; otherwise, it was considered ‘broad’.

b According to the MOoR framework (Appendix 2), and considering only those overviews that mention overlap in their methods or results section. Percentages are calculated considering the total number of included overviews (n = 193) as the denominator.

### 3.3 CCA assessment under different assumptions

We selected a sample of seven overviews to test the quantitative assessment of overlap using the CCA under different matrix assumptions.^41^^–^ ^46^ Four of these overviews had a narrow scope,^43^^–^ ^46^ and three had a broad scope.^41^^,^ ^42^^,^ ^47^ The number of included SRs in each overview ranged from five^41^ to 51.^46^ Appendix 5 provides further details about the seven sampled overviews.

**Figure 2:**
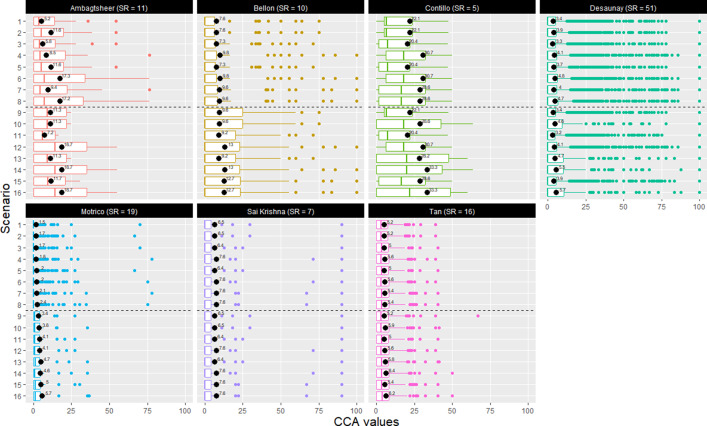
Box plots showing overall and pairwise CCA values for each overview, according to predefined scenarios. This figure summarises the overall and pairwise CCA for each scenario per overview. Each facet presents the result for a specific overview, with each overview author’s name (and the number of included SRs in the overview) in the header. The X-axis contains the CCA value, expressed as a percentage. The Y-axis contains each one of the predefined scenarios (see Table 2). The black dots and the value on the right of each dot indicate the overall CCA. The box plot represents a summary of the pairwise CCA values. The dashed line in each facet separates the overview-level scenarios (1–8) from the outcome-level scenarios (9–16). CCA: corrected covered area; SR: systematic review.

We constructed 16 matrices for each overview, according to the scenarios outlined in Table 2. Figure 2 visually summarises the overall and pairwise CCA assessments for each overview and scenario. As expected, scenario 1 usually shows the lowest CCA values, whereas scenario 16 shows the highest CCA values.

In our sample, the potential impact on the overall CCA approach (i.e. the difference between the maximum and minimum value) ranged from 1.2%^43^ to 13.5%.^47^ The potential impact of the pairwise approach ranged from 0.0%^43^^,^ ^45^^,^ ^46^ to 15.7%.^41^ Appendix 6 details the range between minimum and maximum CCA values for each overview and scenario.

## 4 Discussion

Among the 193 overviews of SRs of health interventions identified in this methodological review, almost half (44.0%) did not mention overlap, and a few (7.3%) mentioned it only in the discussion. Nearly half (48.7%) of the overviews explicitly accounted for overlap within the methods or results, and in those studies, it was most often addressed in the synthesis and summary of findings step. Most had a broad scope (66.8%), and only 11.9% used an overview-specific reporting guideline when submitting their manuscript for publication. This limited adoption of overview-specific reporting guidelines (e.g. PRIOR) alongside the reliance on SR-specific guidelines (e.g. PRISMA) may be partly explained by the recency of these tools, with PRIOR—widely considered the most recommended guideline for overviews of health interventions—being published in August 2022.^21^

We conducted a detailed *de novo* CCA assessment for a subset of seven overviews, using 16 predefined scenarios. These scenarios represented different assumptions depending on the level of analysis—overview level (scenarios 1–8) and outcome level (scenarios 9–16)—and three methodological assumptions, namely scope, publication-thread, and structural missingness. For each scenario, we calculated both overall and pairwise CCA. As expected, matrices adjusted with these three assumptions usually exhibited higher CCA values, as the adjustments removed ‘noise’ (i.e. blank intersections). For example, a matrix adjusted by scope deleted the rows of the primary studies that did not contribute data for an overview outcome, and it is more likely for these specific primary studies to be included only in one (or few) SRs within the overview.

We found that the maximum potential impact of selecting different scenarios on CCA ranged from 1.2% to 13.5% for overall CCA, and 0.0% to 15.7% for pairwise CCA. Level of analysis, scope adjustment, and structural missingness adjustment had the greatest impact on these variations, whereas publication-thread adjustments had little effect on CCA in our sample. Despite these general ideas, it is worth discussing specific aspects identified for each overview.

Of the sampled overviews, Ambagtsheer and Contillo showed the greatest variability of CCA depending on different adjustment scenarios.^38^^,^ ^44^ In Contillo’s overview—which included the fewest references per SR (mean = 5.2)—the unadjusted scenario produced an overall CCA of 22.1%. Adjusting for structural missingness caused the largest shifts in CCA values, whereas scope adjustment (scenario 2) left the overall CCA unchanged from the unadjusted value, and publication-thread adjustment (scenario 3) yielded only minimal differences. Moreover, this overview consistently exhibited a higher overall CCA than its median pairwise CCA, underscoring the importance of using both overall and pairwise measures to accurately describe overlap in overviews.^11^

Ambagtsheer’s overview similarly highlights how scenario choice can influence CCA interpretation, though with markedly different results.^44^ This analysis has the highest reference density of all sampled overviews (mean = 22.2 references per SR) yet yields a much lower unadjusted overall CCA of just 5.2%. Unlike in Contillo’s overview, Ambagtsheer’s CCA values were driven chiefly by scope adjustments: when recalculated under scenario 2 (scope adjusted), the CCA increased by 6.4% compared with the non-adjusted scenario 1, a moderate change. Interestingly, when we limit the analysis to the outcome-level scope, adjustments have minimal impact—the overall CCA stays at 11.3% under both scenarios 9 and 10. In contrast, accounting for structural missingness moderately increases overlap, raising the CCA from 11.3% (scenario 9) to 18.7% (scenario 12). Because outcome-level analyses include only reviews with data on that outcome, changing the review scope does little to alter overlap, whereas filling in missing structural links reveals considerable additional duplication. Since outcome-level analysis includes only reviews with data on the specific outcome, scope adjustments are unlikely to significantly affect CCA. On the other hand, structural missingness adjustments may be an important factor explaining the variability of the CCA, reflecting a major problem related to the inclusion of outdated SRs in overviews, which may compromise the comparability of different SRs analysing the same phenomenon.^45^

The overview by Tan showed minimal variations in overall CCA (1.4%) and median pairwise CCA (0.6%) across scenarios.^41^ This overview included 16 SRs with a mean of 11.4 primary studies per review, but had a narrow scope. Indeed, according to our subjective assessment, we think this overview had the narrowest scope among those sampled, which could explain the stability of the CCA across different scenarios.

In the overviews by Bellon, Desaunay, Motrico, and Sai Krishna, changing the matrix-construction assumptions led to led to minimal changes in overall CCA in most cases (≤5%), with Bellon’s overview showing a moderate change of 5.7%.^39^^,^ ^40^^,^ ^42^^,^ ^43^ Yet in every case, the pairwise CCA stayed at 0.0% across all scenarios. This pattern shows that, although the total amount of duplicated study entries can vary slightly depending on how the matrix is built, virtually no individual SR-to-SR comparison actually shares any primary studies, so pairwise overlap remains zero. In the Desaunay panel of Figure 2, the median pairwise CCA stays at 0.0% across all scenarios, showing that most SR pairs share no primary studies. However, a handful of outliers reveal specific review pairs with markedly different overlap, highlighting how these few cases can disproportionately affect interpretation despite the generally low overall CCA.

The implications of overlap across SRs are context-dependent, subject to interpretation, and not inherently positive or negative. High overlap may introduce bias and distort the precision of the results by giving disproportionate influence to certain primary studies, and may be considered research waste, as redundant reviews consume resources without necessarily adding new knowledge. At the same time, overlap can represent legitimate scientific replication, and when overlapping reviews—despite using different methods or eligibility criteria—reach consistent conclusions, this may strengthen confidence in the robustness of findings for the interventions under study.^48^ Conversely, low overlap coupled with heterogeneous results may signal that conclusions are highly sensitive to differences in eligibility criteria or scope of the reviews, potentially reflecting a broad overview scope, thereby requiring cautious interpretation. Thus, overlap should be considered both as a methodological challenge and as a potentially informative feature of the evidence landscape.

Currently, creating evidence matrices is highly burdensome. Most SRs do not separate the citations of included primary studies from other references—instead, journals usually list all sources together (with the remarkable exception of Cochrane), making it very labour-intensive to identify which studies were actually analysed. Constructing a single matrix—including all different scenarios—required more than one week of full-time work, which limited our capacity to include more than seven overviews in the present sample. As far as we are aware, there is no metadata signalling which references corresponds to the included studies in a SR. Addressing this gap in the future could greatly facilitate the identification of primary studies and support meta-epidemiological research in evidence synthesis. In addition, we expect that explicitly considering and reporting the matrix-construction assumptions before starting will help authors define the most appropriate scenarios in advance, thereby reducing unnecessary work and increasing both efficiency and the clinical usefulness of the resulting matrices.

Another limitation is that authors often overlook publication-thread issues. Multiple papers reporting on the same primary study population pose a significant challenge in medical publishing, and to date, there is no universally accepted editorial solution.^27^^,^ ^49^^–^ ^51^ Some SR authors implicitly adjust for publication-thread by citing only one article per study, which may explain why publication-thread adjustments had no significant impact on CCA in our sample. Nevertheless, in settings where multiple references per study are common, this adjustment could substantially influence overlap estimates.

This is the first study exploring the different assumptions for constructing matrices of evidence on a quantitative overlap assessment. Our review highlights key challenges that persist in overview reporting and overlap assessment, including the limited reporting of overlap, underuse of overview-specific guidelines, inconsistent integration of overlap across methodological steps, and considerable variability in overlap estimates depending on analytical assumptions—several of which have also been noted in previous studies.^5^^,^ ^10^^–^ ^12^^,^ ^52^ This raises questions about the validity of existing CCA thresholds—low (<5%), moderate (5–10%), high (10–15%), and very high (≥15%)—used to describe overlap.^2^^,^ ^16^ We recommend applying these cut-offs with caution because (i) they were proposed arbitrarily and have not been tested in methodological studies to understand their impact; and (ii) as our review demonstrates, CCA values can vary depending on the assumptions made when constructing the evidence matrix. Therefore, any methodological recommendations based on CCA results should be refined and validated.^36^ Our definitions serve as an initial guidance for improving the reporting of evidence matrix construction and, therefore, quantitative overlap assessment through CCA.

## 5 Conclusion

We offer preliminary guidance for transparently reporting evidence matrix construction, which will, in turn, enhance the accuracy and reproducibility of quantitative overlap assessments using CCA. In conclusion, our analysis reveals that the way an evidence matrix is constructed—through decisions about rows versus columns, scope definitions, structural missingness, and publication‐thread handling—significantly alters CCA values. This variability undermines uncritical use of the current overlap thresholds (<5% low, 5–10% moderate, 10–15% high, ≥15% very high) without empirical validation. To strengthen overlap assessment, we must adopt a transparent, standardised framework that clearly documents matrix construction choices and reports both overall and pairwise CCA measures. By refining CCA protocols in this way, overview authors will be better equipped to synthesise evidence accurately, interpret redundancy appropriately, and make informed methodological decisions in support of robust, evidence-informed healthcare. It is also important to emphasise that CCA was not developed to define the presence or absence of overlap—since some degree of overlap is expectable among reviews with similar scope—but rather to provide an approximate, descriptive summary of its magnitude. While the formula makes it reproducible and robust across approaches, CCA should not be interpreted as a test statistic with fixed thresholds or cut-offs of significance. Instead, it should be applied cautiously as a guide to understanding redundancy in the evidence base.

## Supporting information

Bracchiglione et al. supplementary material Bracchiglione et al. supplementary material

## Data Availability

The protocol for this study was prospectively registered, and it is publicly available at https://doi.org/10.1101/2023.06.16.23291488.^17^ All the supporting data related to this study are publicly available at https://osf.io/hbf5j/.^53^

## Supplementary material

To view supplementary material for this article, please visit http://doi.org/10.1017/rsm.2025.10056.
