# Supplementary material for: Impact of matrix-construction assumptions on quantitative overlap assessment in overviews: A meta-research study
Source: Res Synth Methods. 2025 Nov 17;17(2):348–64. doi: 10.1017/rsm.2025.10056 (PMC12873615; doi:10.1017/rsm.2025.10056)
Supplement: Bracchiglione et al. supplementary material [file S1759287925100562sup001.docx]

## Appendix 1. Search strategy

**MEDLINE/Pubmed**

| Search strategy | ((("overview*"[Title] OR "review"[Title] OR "synthesis"[Title] OR "summary"[Title] OR "cochrane"[Title] OR "analysis"[Title]) AND ("reviews"[Title] OR "meta-analyses"[Title] OR "articles"[Title] OR "umbrella"[Title])) OR "umbrella review"[Title/Abstract] OR ("meta-review"[Title/Abstract] OR "metareview"[Title/Abstract])) AND (2023/1/1:2023/4/30[pdat]) |
| --- | --- |
| Total hits | 682 |

**Cochrane Library**

| Search strategy | "#1 - overview:ti,ab or "network meta-analysis":ti,ab" with Cochrane Library publication date Between Jan 2023 and Apr 2023, in Cochrane Reviews |
| --- | --- |
| Total hits | 8 |

##

## Appendix 2. Methods to address overlap according to the ‘Methods for Overviews of Reviews’ (MOoR) framework

| **Step in the conduct of the overview** | **Methods to deal with overlap** |
| --- | --- |
| **Eligibility criteria** | Include all reviews |
|  | Select one (or more) review according to pre-specified eligibility criteria |
|  | Select one review from multiple addressing the same question using pre-specified decision rules |
|  | In case of multiple reviews available, exclude those not containing any unique primary studies |
| **Data extraction** | Extract all reviews |
|  | Extract data from only one (or more) review according to pre-specified criteria |
| **Assessment of risk of bias step** | Select one (or more) high quality review, or exclude low-quality reviews, according to pre-specified criteria |
| **Synthesis and presentation and summary of findings step** | Quantifying the amount of overlap (e.g. CCA) |
|  | Visually present overlap (e.g. matrix) |
|  | Select one review (e.g. high-quality review using decision rules) |
|  | Use a statistical method (e.g. sensitivity analyses) |

Adapted from Lunny C, Pieper D, Thabet P, et al. Managing overlap of primary study results across systematic reviews: practical considerations for authors of overviews of reviews. BMC Med Res Methodol 2021;21:140. doi:10.1186/s12874-021-01269-y

##

## Appendix 3. List of excluded studies, with reasons

| **Reference** | **Reason for exclusion** |
| --- | --- |
| Aburas S, Pfaffeneder-Mantai F, Hofmann A, Meller O, Schneider B, Turhani D. Dentophobia and dental treatment: An umbrella review of the published literature. Spec Care Dentist [Internet]. 2023;43(2):163–73. Available from: https://www.ncbi.nlm.nih.gov/pubmed/35700448 | Overview not focused on effects of health interventions |
| Ahern S, Walsh KA, Paone S, Browne J, Carrigan M, Harrington P, et al. Clinical efficacy and effectiveness of alternative varicella vaccination strategies: An overview of reviews. Rev Med Virol [Internet]. 2023;33(1):e2407. Available from: http://dx.doi.org/10.1002/rmv.2407 | Not an overview |
| Ahern S, Walsh KA, Paone S, Browne J, Carrigan M, Harrington P, et al. Safety of varicella vaccination strategies: An overview of reviews. Rev Med Virol [Internet]. 2023;33(2):e2416. Available from: http://dx.doi.org/10.1002/rmv.2416 | Overview not focused on effects of health interventions |
| Al-Aqeel S, Alsugair J, Alghamdi R. Economic evaluation of interventions to improve medication adherence among patients with chronic diseases: an overview of systematic reviews. Expert Rev Pharmacoecon Outcomes Res [Internet]. 2023;23(2):153–79. Available from: https://www.ncbi.nlm.nih.gov/pubmed/36562404 | Overview not focused on effects of health interventions |
| Anand Prakash A, K M, Akilesh V. Umbrella review of musculoskeletal injury burden in dancers: implication for practice and research. Phys Sportsmed [Internet]. 2023;1–14. Available from: https://www.ncbi.nlm.nih.gov/pubmed/36757080 | Overview not focused on effects of health interventions |
| Araújo ALD, Santos-Silva AR, Kowalski LP. Diagnostic Accuracy of Liquid Biopsy for Oral Potentially Malignant Disorders and Head and Neck Cancer: an Overview of Systematic Reviews. Curr Oncol Rep [Internet]. 2023;25(4):279–92. Available from: http://dx.doi.org/10.1007/s11912-023-01365-w | Overview not focused on effects of health interventions |
| Arienti C, Patrini M, Negrini S, Kiekens C. Overview of Cochrane Systematic Reviews for Rehabilitation Interventions in Persons With Spinal Cord Injury: A Mapping Synthesis. Arch Phys Med Rehabil [Internet]. 2023;104(1):143–50. Available from: https://www.ncbi.nlm.nih.gov/pubmed/35905770 | Overview not focused on effects of health interventions |
| Arts J, Drotos E, Singh AS, Chinapaw MJM, Altenburg TM, Gubbels JS. Correlates of Physical Activity in 0- to 5-year-olds: A Systematic Umbrella Review and Consultation of International Researchers. Sports Med [Internet]. 2023;53(1):215–40. Available from: http://dx.doi.org/10.1007/s40279-022-01761-5 | Overview not focused on effects of health interventions |
| Asgary S, Eghbal MJ. Challenging the Misnomer of Irreversible Pulpitis and Deliberating the Urgent Need for Reclassification of Pulpal Diseases Based on the Efficacy of Vital Pulp Therapies: An Overview of Systematic Reviews. Iran Endod J [Internet]. 2023;18(4):202–5. Available from: http://dx.doi.org/10.22037/iej.v18i4.43035 | Not an overview |
| Autaubo J, Fitts AB, Wise A, Flores H, Kee M, Garrett M, et al. Assessing the Reporting and Frequency of Harms in Systematic Reviews Focused on Minimally Invasive Hysterectomies: A Cross-sectional Analysis. J Minim Invasive Gynecol [Internet]. 2023;30(2):100–7. Available from: https://www.ncbi.nlm.nih.gov/pubmed/36442755 | Overview not focused on effects of health interventions |
| Avau B, O D, Veys K, Georgsen J, Nahirniak S, Shehata N, et al. Systematic reviews on platelet transfusions: Is there unnecessary duplication of effort? A scoping review. Vox Sang [Internet]. 2023;118(1):16–23. Available from: http://dx.doi.org/10.1111/vox.13387 | Overview not focused on effects of health interventions |
| Ayorinde A, Ghosh I, Ali I, Zahair I, Olarewaju O, Singh M, et al. Health inequalities in infectious diseases: a systematic overview of reviews. BMJ Open [Internet]. 2023;13(4):e067429. Available from: http://dx.doi.org/10.1136/bmjopen-2022-067429 | Overview not focused on effects of health interventions |
| Badran S, Doi SA, Hamdi M, Hammouda A, Alharami S, Clark J, et al. Metabolic aspects of surgical subcutaneous fat removal: An umbrella review and implications for future research. Biomol Biomed [Internet]. 2023;23(2):235–47. Available from: http://dx.doi.org/10.17305/bjbms.2022.8175 | Overview not focused on effects of health interventions |
| Bahramy A, Zafari N, Rajabi F, Aghakhani A, Jayedi A, Khaboushan AS, et al. Prognostic and diagnostic values of non-coding RNAs as biomarkers for breast cancer: An umbrella review and pan-cancer analysis. Front Mol Biosci [Internet]. 2023;10:1096524. Available from: http://dx.doi.org/10.3389/fmolb.2023.1096524 | Overview not focused on effects of health interventions |
| Bai X, Ding SQ, Zhang XP, Han MH, Dai DQ. Exposure to Commonly Used Drugs and the Risk of Gastric Cancer: An Umbrella Review of Meta-Analyses. Cancers [Internet]. 2023;15(2). Available from: http://dx.doi.org/10.3390/cancers15020372 | Overview not focused on effects of health interventions |
| Barreto JOM, Romão DMM, Setti C, Machado MLT, Riera R, Gomes R, et al. Competency profiles for evidence-informed policy-making (EIPM): a rapid review. Health Res Policy Syst [Internet]. 2023;21(1):16. Available from: http://dx.doi.org/10.1186/s12961-023-00964-0 | Overview not focused on effects of health interventions |
| Black L, Panayiotou M, Humphrey N. Measuring general mental health in early-mid adolescence: A systematic meta-review of content and psychometrics. JCPP Adv [Internet]. 2023;3(1):e12125. Available from: http://dx.doi.org/10.1002/jcv2.12125 | Overview not focused on effects of health interventions |
| Bonaccorsi G, Milani C, Giorgetti D, Setola N, Naldi E, Manzi F, et al. Impact of Built Environment and Neighborhood on Promoting Mental Health, Well-being, and Social Participation in Older People: an Umbrella Review. Ann Ig [Internet]. 2023;35(2):213–39. Available from: http://dx.doi.org/10.7416/ai.2022.2534 | Not an overview |
| Bower M, Smout S, Donohoe-Bales A, O’Dean S, Teesson L, Boyle J, et al. A hidden pandemic? An umbrella review of global evidence on mental health in the time of COVID-19. Front Psychiatry [Internet]. 2023;14:1107560. Available from: http://dx.doi.org/10.3389/fpsyt.2023.1107560 | Overview not focused on effects of health interventions |
| Bowman-Perrott L, Gilson C, Boon RT, Ingles KE. Peer-Mediated Interventions for Students with Intellectual and Developmental Disabilities: A Systematic Review of Reviews of Social and Behavioral Outcomes. Dev Neurorehabil [Internet]. 2023;26(2):134–54. Available from: https://www.ncbi.nlm.nih.gov/pubmed/36892164 | Not an overview |
| Cant R, Ryan C, Kelly MA. Use and Effectiveness of Virtual Simulations in Nursing Student Education: An Umbrella Review. Comput Inform Nurs [Internet]. 2023;41(1):31–8. Available from: https://www.ncbi.nlm.nih.gov/pubmed/35926237 | Overview not focused on effects of health interventions |
| Carpallo-González M, Muñoz-Navarro R, González-Blanch C, Cano-Vindel A. Symptoms of emotional disorders and sociodemographic factors as moderators of dropout in psychological treatment: A meta-review. Int J Clin Health Psychol [Internet]. 2023;23(4):100379. Available from: http://dx.doi.org/10.1016/j.ijchp.2023.100379 | Overview not focused on effects of health interventions |
| Carpiniello B. The Mental Health Costs of Armed Conflicts-A Review of Systematic Reviews Conducted on Refugees, Asylum-Seekers and People Living in War Zones. Int J Environ Res Public Health [Internet]. 2023;20(4). Available from: http://dx.doi.org/10.3390/ijerph20042840 | Overview not focused on effects of health interventions |
| Charitakis E, Dragioti E, Stratinaki M, Korela D, Tzeis S, Almroth H, et al. Predictors of recurrence after catheter ablation and electrical cardioversion of atrial fibrillation: an umbrella review of meta-analyses. Europace [Internet]. 2023;25(1):40–8. Available from: http://dx.doi.org/10.1093/europace/euac143 | Not an overview |
| Chatzidimitriou K, Papaioannou W, Seremidi K, Bougioukas K, Haidich AB. Prevalence and association of gastroesophageal reflux disease and dental erosion: An overview of reviews. J Dent [Internet]. 2023;133:104520. Available from: https://www.ncbi.nlm.nih.gov/pubmed/37068653 | Overview not focused on effects of health interventions |
| Chen J, Ke K, Liu Z, Yang L, Wang L, Zhou J, et al. Body Mass Index and Cancer Risk: An Umbrella Review of Meta-Analyses of Observational Studies. Nutr Cancer [Internet]. 2023;75(4):1051–64. Available from: https://www.ncbi.nlm.nih.gov/pubmed/37139871 | Overview not focused on effects of health interventions |
| Chen Y, Hou L, Li Y, Lou Y, Li W, Struble LM, et al. Barriers and motivators to promotion of physical activity participation for older adults with mild cognitive impairment or dementia: An umbrella review. Int J Nurs Stud [Internet]. 2023;143:104493. Available from: http://dx.doi.org/10.1016/j.ijnurstu.2023.104493 | Not an overview |
| Chmielowska M, Zisman-Ilani Y, Saunders R, Pilling S. Trends, challenges, and priorities for shared decision making in mental health: The first umbrella review. Int J Soc Psychiatry [Internet]. 2023;69(4):823–40. Available from: http://dx.doi.org/10.1177/00207640221140291 | Overview not focused on effects of health interventions |
| Choo PY, Shaik MA, Tan-Ho G, Lee J, Ho AHY. Living losses in stroke caregiving: A qualitative systematic review of systematic reviews on psycho-socio-emotional challenges and coping mechanisms. Int J Stroke [Internet]. 2023;18(3):268–77. Available from: http://dx.doi.org/10.1177/17474930221104908 | Overview not focused on effects of health interventions |
| Colaprico C, Addari S, La Torre G. The effects of bullying on healthcare workers: an umbrella review of systematic reviews and meta-analyses. Riv Psichiatr [Internet]. 2023;58(2):41–9. Available from: http://dx.doi.org/10.1708/4022.39973 | Overview not focused on effects of health interventions |
| Colenso-Semple LM, D’Souza AC, Elliott-Sale KJ, Phillips SM. Current evidence shows no influence of women’s menstrual cycle phase on acute strength performance or adaptations to resistance exercise training. Front Sports Act Living [Internet]. 2023;5:1054542. Available from: http://dx.doi.org/10.3389/fspor.2023.1054542 | Overview not focused on effects of health interventions |
| Corôa RC, Gogovor A, Ben Charif A, Hassine AB, Zomahoun HTV, McLean RKD, et al. Evidence on Scaling in Health and Social Care: An Umbrella Review. Milbank Q [Internet]. 2023;101(3):881–921. Available from: http://dx.doi.org/10.1111/1468-0009.12649 | Overview not focused on effects of health interventions |
| Costanzo E, Lengyel I, Parravano M, Biagini I, Veldsman M, Badhwar A, et al. Ocular Biomarkers for Alzheimer Disease Dementia: An Umbrella Review of Systematic Reviews and Meta-analyses. JAMA Ophthalmol [Internet]. 2023;141(1):84–91. Available from: https://www.ncbi.nlm.nih.gov/pubmed/36394831 | Overview not focused on effects of health interventions |
| Croce L, Chiovato L, Tonacchera M, Petrosino E, Tanda ML, Moleti M, et al. Iodine status and supplementation in pregnancy: an overview of the evidence provided by meta-analyses. Rev Endocr Metab Disord [Internet]. 2023;24(2):241–50. Available from: http://dx.doi.org/10.1007/s11154-022-09760-7 | Not an overview |
| Cuijpers P, Miguel C, Ciharova M, Kumar M, Brander L, Kumar P, et al. Impact of climate events, pollution, and green spaces on mental health: an umbrella review of meta-analyses. Psychol Med [Internet]. 2023;53(3):638–53. Available from: http://dx.doi.org/10.1017/S0033291722003890 | Not an overview |
| Dalbøge A, Albert Kolstad H, Ulrik CS, Sherson DL, Meyer HW, Ebbehøj N, et al. The Relationship Between Potential Occupational Sensitizing Exposures and Asthma: An Overview of Systematic Reviews. Ann Work Expo Health [Internet]. 2023;67(2):163–81. Available from: http://dx.doi.org/10.1093/annweh/wxac074 | Overview not focused on effects of health interventions |
| Desmoulin A, Joly E, Tran P, Derancourt C, Bertolotti A. Methodological quality of systematic reviews of the local management of anogenital warts: a systematic review using AMSTAR II, ROBIS and PRISMA. Sex Transm Infect [Internet]. 2023;99(5):345–7. Available from: https://www.ncbi.nlm.nih.gov/pubmed/36948589 | Overview not focused on effects of health interventions |
| Dragioti E, Radua J, Solmi M, Gosling CJ, Oliver D, Lascialfari F, et al. Impact of mental disorders on clinical outcomes of physical diseases: an umbrella review assessing population attributable fraction and generalized impact fraction. World Psychiatry [Internet]. 2023;22(1):86–104. Available from: http://dx.doi.org/10.1002/wps.21068 | Overview not focused on effects of health interventions |
| Duong KNC, Le LM, Veettil SK, Saidoung P, Wannaadisai W, Nelson RE, et al. Disparities in COVID-19 related outcomes in the United States by race and ethnicity pre-vaccination era: an umbrella review of meta-analyses. Front Public Health [Internet]. 2023;11:1206988. Available from: http://dx.doi.org/10.3389/fpubh.2023.1206988 | Overview not focused on effects of health interventions |
| Farazi M, Jayedi A, Shab-Bidar S. Dietary inflammatory index and the risk of non-communicable chronic disease and mortality: an umbrella review of meta-analyses of observational studies. Crit Rev Food Sci Nutr [Internet]. 2023;63(1):57–66. Available from: https://www.ncbi.nlm.nih.gov/pubmed/34176394 | Overview not focused on effects of health interventions |
| Félix J, Moreira J, Santos R, Kontio E, Pinheiro AR, Sousa ASP. Health-Related Telemonitoring Parameters/Signals of Older Adults: An Umbrella Review. Sensors [Internet]. 2023;23(2). Available from: http://dx.doi.org/10.3390/s23020796 | Overview not focused on effects of health interventions |
| Feng W, Wang X, Huang D, Lu A. Role of diet in osteoporosis incidence: Umbrella review of meta-analyses of prospective observational studies. Crit Rev Food Sci Nutr [Internet]. 2023;63(19):3420–9. Available from: https://www.ncbi.nlm.nih.gov/pubmed/34644187 | Overview not focused on effects of health interventions |
| Fernandes TO, Carvalho PA, Abreu FV, Kirschneck C, Küchler EC, Antunes LS, et al. Association between nutritional status and children and adolescents’ dental caries experiences: an overview of systematic reviews. J Appl Oral Sci [Internet]. 2023;31:e20230138. Available from: http://dx.doi.org/10.1590/1678-7757-2023-0138 | Overview not focused on effects of health interventions |
| Fouladi N, Tchangalova N, Ajayi D, Millwee E, Lovett C, Del Sordi A, et al. COVID-19 Public Health Measures and Patient and Public Involvement in Health and Social Care Research: An Umbrella Review. Int J Environ Res Public Health [Internet]. 2023;20(6). Available from: http://dx.doi.org/10.3390/ijerph20064887 | Not an overview |
| Gianola S, Bargeri S, Biffi A, Cimbanassi S, D’Angelo D, Coclite D, et al. Structured approach with primary and secondary survey for major trauma care: an overview of reviews. World J Emerg Surg [Internet]. 2023;18(1):2. Available from: http://dx.doi.org/10.1186/s13017-022-00472-6 | Overview not focused on effects of health interventions |
| Gilles C, Konopnicki D, Rozenberg S. The recent natural history of human papillomavirus cervical infection in women living with HIV: A scoping review of meta-analyses and systematic reviews and the construction of a hypothetical model. HIV Med [Internet]. 2023;24(8):877–92. Available from: https://www.ncbi.nlm.nih.gov/pubmed/37062862 | Overview not focused on effects of health interventions |
| Gillespie SM, Jones A, Garofalo C. Psychopathy and dangerousness: An umbrella review and meta-analysis. Clin Psychol Rev [Internet]. 2023;100:102240. Available from: https://www.ncbi.nlm.nih.gov/pubmed/36608488 | Overview not focused on effects of health interventions |
| González-Palacios Torres C, Barrios-Rodríguez R, Muñoz-Bravo C, Toledo E, Dierssen T, Jiménez-Moleón JJ. Mediterranean diet and risk of breast cancer: An umbrella review. Clin Nutr [Internet]. 2023;42(4):600–8. Available from: http://dx.doi.org/10.1016/j.clnu.2023.02.012 | Overview not focused on effects of health interventions |
| Guccione L, Fullerton S, Gough K, Hyatt A, Tew M, Aranda S, et al. Why is advance care planning underused in oncology settings? A systematic overview of reviews to identify the benefits, barriers, enablers, and interventions to improve uptake. Front Oncol [Internet]. 2023;13:1040589. Available from: http://dx.doi.org/10.3389/fonc.2023.1040589 | Not an overview |
| Hazzard VM, Mason TB, Smith KE, Schaefer LM, Anderson LM, Dodd DR, et al. Identifying transdiagnostically relevant risk and protective factors for internalizing psychopathology: An umbrella review of longitudinal meta-analyses. J Psychiatr Res [Internet]. 2023;158:231–44. Available from: https://www.ncbi.nlm.nih.gov/pubmed/36603318 | Overview not focused on effects of health interventions |
| Hernigou P, Barbier O, Chenaie P. Hip arthroplasty dislocation risk calculator: evaluation of one million primary implants and twenty-five thousand dislocations with deep learning artificial intelligence in a systematic review of reviews. Int Orthop [Internet]. 2023;47(2):557–71. Available from: https://www.ncbi.nlm.nih.gov/pubmed/36445413 | Overview not focused on effects of health interventions |
| Hickey L, Kuyucak O, Clausen L, Imms C. Mapping the focus of research conducted with adults with cerebral palsy: an overview of systematic reviews. Disabil Rehabil [Internet]. 2023;45(2):185–208. Available from: https://www.ncbi.nlm.nih.gov/pubmed/35114857 | Overview not focused on effects of health interventions |
| Hogg B, Gardoki-Souto I, Valiente-Gómez A, Rosa AR, Fortea L, Radua J, et al. Psychological trauma as a transdiagnostic risk factor for mental disorder: an umbrella meta-analysis. Eur Arch Psychiatry Clin Neurosci [Internet]. 2023;273(2):397–410. Available from: http://dx.doi.org/10.1007/s00406-022-01495-5 | Overview not focused on effects of health interventions |
| Howard H, Clark P, Garrett M, Wise A, Kee M, Checketts J, et al. A cross-sectional analysis of harms reporting in systematic reviews evaluating laminectomy. Spine J [Internet]. 2023;13:100198. Available from: http://dx.doi.org/10.1016/j.xnsj.2022.100198 | Overview not focused on effects of health interventions |
| Hua J, Liu J, Ma M, Xie L, Tian J. MicroRNA in the diagnosis of lung cancer: An overview of ten systematic reviews. Ann Clin Biochem [Internet]. 2023;60(1):6–13. Available from: http://dx.doi.org/10.1177/00045632221128684 | Overview not focused on effects of health interventions |
| Huang Y, Cao D, Chen Z, Chen B, Li J, Wang R, et al. Iron intake and multiple health outcomes: Umbrella review. Crit Rev Food Sci Nutr [Internet]. 2023;63(16):2910–27. Available from: https://www.ncbi.nlm.nih.gov/pubmed/34583608 | Overview not focused on effects of health interventions |
| Huang Y, Chen Z, Chen B, Li J, Yuan X, Wang W, et al. Dietary sugar consumption and health: umbrella review. BMJ [Internet]. 2023;381:e071609. Available from: http://dx.doi.org/10.1136/bmj-2022-071609 | Overview not focused on effects of health interventions |
| Huggard L, Murphy R, O’Connor C, Nearchou F. The Social Determinants of Mental Illness: A Rapid Review of Systematic Reviews. Issues Ment Health Nurs [Internet]. 2023;44(4):302–12. Available from: https://www.ncbi.nlm.nih.gov/pubmed/36972547 | Overview not focused on effects of health interventions |
| Huggins M, Pesut B, Puurveen G. Interventions for Caregivers of Older Adults with Dementia Living in the Community: A Rapid Review of Reviews. Can J Aging [Internet]. 2023;42(3):425–33. Available from: http://dx.doi.org/10.1017/S0714980823000016 | Overview not focused on effects of health interventions |
| Hughes K, Ford H, Thangaratinam S, Brennecke S, Mol BW, Wang R. Diagnosis or prognosis? An umbrella review of mid-trimester cervical length and spontaneous preterm birth. BJOG [Internet]. 2023;130(8):866–79. Available from: http://dx.doi.org/10.1111/1471-0528.17443 | Overview not focused on effects of health interventions |
| Hume S, Brown SR, Mahtani KR. School closures during COVID-19: an overview of systematic reviews. BMJ Evid Based Med [Internet]. 2023;28(3):164–74. Available from: http://dx.doi.org/10.1136/bmjebm-2022-112085 | Overview not focused on effects of health interventions |
| Huo YJ, Li XY, Zhang M, Gao C, Xiao Q, Zhao YH, et al. Strong Cumulative Evidence of Associations of 6 Single Nucleotide Polymorphisms with Ovarian Cancer Risk: An Umbrella Review. J Clin Med Res [Internet]. 2023;12(5). Available from: http://dx.doi.org/10.3390/jcm12052025 | Overview not focused on effects of health interventions |
| Jabbari M, Eini-Zinab H, Safaei E, Poursoleiman F, Amini B, Babashahi M, et al. Determination of the level of evidence for the association between different food groups/items and dietary fiber intake and the risk of cardiovascular diseases and hypertension: An umbrella review. Nutr Res [Internet]. 2023;111:1–13. Available from: https://www.ncbi.nlm.nih.gov/pubmed/36780863 | Not an overview |
| Jay MA, Sanders-Ellis D, Blackburn R, Deighton J, Gilbert R. Umbrella systematic review finds limited evidence that school absence explains the association between chronic health conditions and lower academic attainment. Front Public Health [Internet]. 2023;11:1122769. Available from: http://dx.doi.org/10.3389/fpubh.2023.1122769 | Overview not focused on effects of health interventions |
| Jenabi E, Ayubi E, Khazaei S, Soltanian AR, Salehi AM. The environmental risk factors associated with ectopic pregnancy: An umbrella review. J Gynecol Obstet Hum Reprod [Internet]. 2023;52(2):102532. Available from: https://www.ncbi.nlm.nih.gov/pubmed/36592890 | Overview not focused on effects of health interventions |
| Jones G, Hemmerich C, Rucker B, Wise A, Kee M, Johnson A, et al. Harms reporting by systematic reviews for functional endoscopic sinus surgery: a cross-sectional analysis. Eur Arch Otorhinolaryngol [Internet]. 2023;280(6):2805–19. Available from: https://www.ncbi.nlm.nih.gov/pubmed/36595047 | Overview not focused on effects of health interventions |
| Kaka AS, Landsteiner A, Ensrud KE, Logan B, Sowerby C, Ullman K, et al. Risk prediction models for diabetic foot ulcer development or amputation: a review of reviews. J Foot Ankle Res [Internet]. 2023;16(1):13. Available from: http://dx.doi.org/10.1186/s13047-023-00610-6 | Overview not focused on effects of health interventions |
| Khan AM, Ahmed S, Sarfraz Z, Farahmand P. Vaping and Mental Health Conditions in Children: An Umbrella Review. Subst Abuse [Internet]. 2023;17:11782218231167322. Available from: http://dx.doi.org/10.1177/11782218231167322 | Overview not focused on effects of health interventions |
| Kilpatrick K, Tchouaket E, Savard I, Chouinard MC, Bouabdillah N, Provost-Bazinet B, et al. Identifying indicators sensitive to primary healthcare nurse practitioner practice: A review of systematic reviews. PLoS One [Internet]. 2023;18(9):e0290977. Available from: http://dx.doi.org/10.1371/journal.pone.0290977 | Overview not focused on effects of health interventions |
| Kim B, Royle M. Domestic Violence in the Context of the COVID-19 Pandemic: A Synthesis of Systematic Reviews. Trauma Violence Abuse [Internet]. 2023;15248380231155530. Available from: http://dx.doi.org/10.1177/15248380231155530 | Overview not focused on effects of health interventions |
| Kim W, Kim JH, Cha YK, Chong S, Kim TJ. Completeness of Reporting of Systematic Reviews and Meta-Analysis of Diagnostic Test Accuracy (DTA) of Radiological Articles Based on the PRISMA-DTA Reporting Guideline. Acad Radiol [Internet]. 2023;30(2):258–75. Available from: https://www.ncbi.nlm.nih.gov/pubmed/35491344 | Overview not focused on effects of health interventions |
| Kohler S, Dippon L, Helsper N, Rütten A, Abu-Omar K, Birkholz L, et al. Population-based physical activity promotion with a focus on health equity: a review of reviews. Int J Equity Health [Internet]. 2023;22(1):18. Available from: http://dx.doi.org/10.1186/s12939-023-01834-5 | Not an overview |
| La Torre G, Bova R, Cocchiara RA, Sestili C, Tagliaferri A, Maggiacomo S, et al. What Are the Determinants of the Quality of Systematic Reviews in the International Journals of Occupational Medicine? A Methodological Study Review of Published Literature. Int J Environ Res Public Health [Internet]. 2023;20(2). Available from: http://dx.doi.org/10.3390/ijerph20021644 | Overview not focused on effects of health interventions |
| La Torre G, Vitello T, Cocchiara RA, Della Rocca C. Relationship between formaldehyde exposure, respiratory irritant effects and cancers: a review of reviews. Public Health [Internet]. 2023;218:186–96. Available from: http://dx.doi.org/10.1016/j.puhe.2023.03.009 | Overview not focused on effects of health interventions |
| Lee J, Sohn C, Kim OY, Lee YM, Yoon MO, Lee M. The association between dietary sodium intake and obesity in adults by sodium intake assessment methods: a review of systematic reviews and re-meta-analysis. Nutr Res Pract [Internet]. 2023;17(2):175–91. Available from: http://dx.doi.org/10.4162/nrp.2023.17.2.175 | Overview not focused on effects of health interventions |
| Lee S, Lee KH, Park KM, Park SJ, Kim WJ, Lee J, et al. Impact of data extraction errors in meta-analyses on the association between depression and peripheral inflammatory biomarkers: an umbrella review. Psychol Med [Internet]. 2023;53(5):2017–30. Available from: https://www.ncbi.nlm.nih.gov/pubmed/34749836 | Overview not focused on effects of health interventions |
| Lewis K, Hinchcliff R. Hospital accreditation: an umbrella review. Int J Qual Health Care [Internet]. 2023;35(1). Available from: http://dx.doi.org/10.1093/intqhc/mzad007 | Not an overview |
| Li H, Xia J, Bennett D, Roque F, Bam RA, Tavares ABT, et al. Long-COVID-19 clinical and health outcomes: an umbrella review. Ther Adv Infect Dis [Internet]. 2023;10:20499361231198335. Available from: http://dx.doi.org/10.1177/20499361231198335 | Overview not focused on effects of health interventions |
| Li XY, Li G, Gong TT, Lv JL, Gao C, Liu FH, et al. Non-Genetic Factors and Risk of Cervical Cancer: An Umbrella Review of Systematic Reviews and Meta-Analyses of Observational Studies. Int J Public Health [Internet]. 2023;68:1605198. Available from: http://dx.doi.org/10.3389/ijph.2023.1605198 | Overview not focused on effects of health interventions |
| Liguori S, Young VM, Arienti C, Pollini E, Patrini M, Gimigliano F, et al. Overview of Cochrane systematic reviews for rehabilitation interventions in individuals with cerebral palsy: A mapping synthesis. Dev Med Child Neurol [Internet]. 2023;65(10):1280–91. Available from: http://dx.doi.org/10.1111/dmcn.15572 | Not an overview |
| Limbu YB, Gautam RK. The determinants of COVID-19 vaccination intention: a meta-review. Front Public Health [Internet]. 2023;11:1162861. Available from: http://dx.doi.org/10.3389/fpubh.2023.1162861 | Overview not focused on effects of health interventions |
| Liu M, Gao Y, Yang K, Cai Y, Xu J, Dai D, et al. Reporting quality and risk of bias of Cochrane individual participant data meta-analyses: A cross-sectional study. J Evid Based Med [Internet]. 2023;16(2):141–51. Available from: https://www.ncbi.nlm.nih.gov/pubmed/37020358 | Overview not focused on effects of health interventions |
| Liu WY, Jiesisibieke ZL, Chien CW, Tung TH. Association between COVID-19 and sexual health: an umbrella review. Ann Med [Internet]. 2023;55(2):2258902. Available from: http://dx.doi.org/10.1080/07853890.2023.2258902 | Overview not focused on effects of health interventions |
| Liu Z, Al Amer FM, Xiao M, Xu C, Furuya-Kanamori L, Hong H, et al. The normality assumption on between-study random effects was questionable in a considerable number of Cochrane meta-analyses. BMC Med [Internet]. 2023;21(1):112. Available from: http://dx.doi.org/10.1186/s12916-023-02823-9 | Not an overview |
| Majid U, Hussain SAS, Zahid A, Haider MH, Arora R. Mental health outcomes in health care providers during the COVID-19 pandemic: an umbrella review. Health Promot Int [Internet]. 2023;38(2). Available from: http://dx.doi.org/10.1093/heapro/daad025 | Overview not focused on effects of health interventions |
| Mandic M, Li H, Safizadeh F, Niedermaier T, Hoffmeister M, Brenner H. Is the association of overweight and obesity with colorectal cancer underestimated? An umbrella review of systematic reviews and meta-analyses. Eur J Epidemiol [Internet]. 2023;38(2):135–44. Available from: http://dx.doi.org/10.1007/s10654-022-00954-6 | Overview not focused on effects of health interventions |
| Mazza MG, Palladini M, Villa G, Agnoletto E, Harrington Y, Vai B, et al. Prevalence of depression in SARS-CoV-2 infected patients: An umbrella review of meta-analyses. Gen Hosp Psychiatry [Internet]. 2023;80:17–25. Available from: http://dx.doi.org/10.1016/j.genhosppsych.2022.12.002 | Overview not focused on effects of health interventions |
| McCarty DB, Letzkus L, Attridge E, Dusing SC. Efficacy of Therapist Supported Interventions from the Neonatal Intensive Care Unit to Home: A Meta-Review of Systematic Reviews. Clin Perinatol [Internet]. 2023;50(1):157–78. Available from: https://www.ncbi.nlm.nih.gov/pubmed/36868703 | Not an overview |
| McCready JL, Nichol B, Steen M, Unsworth J, Comparcini D, Tomietto M. Understanding the barriers and facilitators of vaccine hesitancy towards the COVID-19 vaccine in healthcare workers and healthcare students worldwide: An Umbrella Review. PLoS One [Internet]. 2023;18(4):e0280439. Available from: http://dx.doi.org/10.1371/journal.pone.0280439 | Overview not focused on effects of health interventions |
| McGrane E, Wardle H, Clowes M, Blank L, Pryce R, Field M, et al. What is the evidence that advertising policies could have an impact on gambling-related harms? A systematic umbrella review of the literature. Public Health [Internet]. 2023;215:124–30. Available from: http://dx.doi.org/10.1016/j.puhe.2022.11.019 | Overview not focused on effects of health interventions |
| Meshkat S, Ho RC, Cao B, Teopiz KM, Rosenblat JD, Rhee TG, et al. Biomarkers of ketamine’s antidepressant effect: An umbrella review. J Affect Disord [Internet]. 2023;323:598–606. Available from: https://www.ncbi.nlm.nih.gov/pubmed/36521662 | Overview not focused on effects of health interventions |
| Mirza AA, Abdulazeem HM, Al-Sayed AA, Alandejani TA, Shawli HY, Thompson JY, et al. Biologic Therapies in Chronic Rhinosinusitis with Nasal Polyposis: Overview of Systematic Reviews and Updated Systematic Review. Indian J Otolaryngol Head Neck Surg [Internet]. 2023;75(Suppl 1):650–60. Available from: http://dx.doi.org/10.1007/s12070-022-03144-8 | Not an overview |
| Mitrogiannis I, Evangelou E, Efthymiou A, Kanavos T, Birbas E, Makrydimas G, et al. Risk factors for preterm labor: An Umbrella Review of meta-analyses of observational studies. Res Sq [Internet]. 2023; Available from: http://dx.doi.org/10.21203/rs.3.rs-2639005/v1 | Overview not focused on effects of health interventions |
| Muka T, Li JJX, Farahani SJ, Ioannidis JPA. An umbrella review of systematic reviews on the impact of the COVID-19 pandemic on cancer prevention and management, and patient needs. Elife [Internet]. 2023;12. Available from: http://dx.doi.org/10.7554/eLife.85679 | Overview not focused on effects of health interventions |
| Najafi F, Rajati F, Sarokhani D, Bavandpour M, Moradinazar M. The Relationship between Metformin Consumption and Cancer Risk: An Updated Umbrella Review of Systematic Reviews and Meta-Analyses. Int J Prev Med [Internet]. 2023;14:90. Available from: http://dx.doi.org/10.4103/ijpvm.ijpvm_62_21 | Overview not focused on effects of health interventions |
| Nichol B, McCready JL, Steen M, Unsworth J, Simonetti V, Tomietto M. Barriers and facilitators of vaccine hesitancy for COVID-19, influenza, and pertussis during pregnancy and in mothers of infants under two years: An umbrella review. PLoS One [Internet]. 2023;18(3):e0282525. Available from: http://dx.doi.org/10.1371/journal.pone.0282525 | Overview not focused on effects of health interventions |
| Núñez-Núñez M, Maes-Carballo M, Mignini LE, Chien PFW, Khalaf Y, Fawzy M, et al. Research integrity in randomized clinical trials: A scoping umbrella review. Int J Gynaecol Obstet [Internet]. 2023;162(3):860–76. Available from: http://dx.doi.org/10.1002/ijgo.14762 | Overview not focused on effects of health interventions |
| Olusanya BO, Smythe T, Ogbo FA, Nair MKC, Scher M, Davis AC. Global prevalence of developmental disabilities in children and adolescents: A systematic umbrella review. Front Public Health [Internet]. 2023;11:1122009. Available from: http://dx.doi.org/10.3389/fpubh.2023.1122009 | Overview not focused on effects of health interventions |
| Padilla-Cáceres T, Arbildo-Vega HI, Caballero-Apaza L, Cruzado-Oliva F, Mamani-Cori V, Cervantes-Alagón S, et al. Association between the Risk of Preterm Birth and Low Birth Weight with Periodontal Disease in Pregnant Women: An Umbrella Review. Dent J [Internet]. 2023;11(3). Available from: http://dx.doi.org/10.3390/dj11030074 | Overview not focused on effects of health interventions |
| Park JM, Woo W, Lee SC, Park S, Yon DK, Lee SW, et al. Prevalence and Mortality Risk of Neurological Disorders during the COVID-19 Pandemic: An Umbrella Review of the Current Evidence. Neuroepidemiology [Internet]. 2023;57(3):129–47. Available from: http://dx.doi.org/10.1159/000530536 | Overview not focused on effects of health interventions |
| Pawils S, Heumann S, Schneider SA, Metzner F, Mays D. The current state of international research on the effectiveness of school nurses in promoting the health of children and adolescents: An overview of reviews. PLoS One [Internet]. 2023;18(2):e0275724. Available from: http://dx.doi.org/10.1371/journal.pone.0275724 | Not an overview |
| Pearce S, Marchand T, Shannon T, Ganshorn H, Lang E. Emergency department crowding: an overview of reviews describing measures causes, and harms. Intern Emerg Med [Internet]. 2023;18(4):1137–58. Available from: http://dx.doi.org/10.1007/s11739-023-03239-2 | Overview not focused on effects of health interventions |
| Peruzzi M, Covi K, Saccucci M, Pingitore A, Saade W, Sciarra L, et al. Current knowledge on the association between cardiovascular and periodontal disease: an umbrella review. Minerva Cardiol Angiol [Internet]. 2023;71(2):208–20. Available from: https://www.ncbi.nlm.nih.gov/pubmed/35332749 | Overview not focused on effects of health interventions |
| Phimphasone-Brady P, Page CE, Ali DA, Haller HC, Duffy KA. Racial and ethnic disparities in women’s mental health: a narrative synthesis of systematic reviews and meta-analyses of the US-based samples. Fertil Steril [Internet]. 2023;119(3):364–74. Available from: https://www.ncbi.nlm.nih.gov/pubmed/36702342 | Overview not focused on effects of health interventions |
| Pinto JO, Peixoto B, Dores AR, Barbosa F. Measures of cognitive reserve: An umbrella review. Clin Neuropsychol [Internet]. 2023;1–74. Available from: https://www.ncbi.nlm.nih.gov/pubmed/37073431 | Overview not focused on effects of health interventions |
| Pinto KP, Serrão G, Alves Ferreira CM, Sassone LM, Fidalgo TKDS, Silva EJNL. Association between Apical Periodontitis and Chronic Diseases: An Umbrella Review. Iran Endod J [Internet]. 2023;18(3):134–44. Available from: http://dx.doi.org/10.22037/iej.v18i3.42560 | Overview not focused on effects of health interventions |
| Qin X, Chen J, Jia G, Yang Z. Dietary Factors and Pancreatic Cancer Risk: An Umbrella Review of Meta-Analyses of Prospective Observational Studies. Adv Nutr [Internet]. 2023;14(3):451–64. Available from: http://dx.doi.org/10.1016/j.advnut.2023.02.004 | Overview not focused on effects of health interventions |
| R K, L S, P B, S G, R LP. Psychosocial experiences of breast cancer survivors: a meta-review. J Cancer Surviv [Internet]. 2023; Available from: http://dx.doi.org/10.1007/s11764-023-01336-x | Overview not focused on effects of health interventions |
| Rahnemayan S, Ahari SG, Rikhtegar R, Riyahifar S, Sanaie S. An umbrella review of systematic reviews with meta-analysis on the role of vitamins in Parkinson’s disease. Acta Neurol Belg [Internet]. 2023;123(1):69–83. Available from: http://dx.doi.org/10.1007/s13760-022-02055-3 | Overview not focused on effects of health interventions |
| Reis N, Gaspar L, Paiva A, Sousa P, Machado N. Effectiveness of Nonpharmacological Interventions in the Field of Ventilation: An Umbrella Review. Int J Environ Res Public Health [Internet]. 2023;20(7). Available from: http://dx.doi.org/10.3390/ijerph20075239 | Not an overview |
| Roberts JA, Croom K, Adomakoh N. Continuous infusion of beta-lactam antibiotics: narrative review of systematic reviews, and implications for outpatient parenteral antibiotic therapy. Expert Rev Anti Infect Ther [Internet]. 2023;21(4):375–85. Available from: http://dx.doi.org/10.1080/14787210.2023.2184347 | Not an overview |
| Roberts N, Carrigan A, Clay-Williams R, Hibbert PD, Mahmoud Z, Pomare C, et al. Innovative models of healthcare delivery: an umbrella review of reviews. BMJ Open [Internet]. 2023;13(2):e066270. Available from: http://dx.doi.org/10.1136/bmjopen-2022-066270 | Overview not focused on effects of health interventions |
| Sabater-Martos M, Martínez-Pastor JC, Morales A, Ferrer M, Antequera A, Roqué M. Overview of systematic reviews of risk factors for prosthetic joint infection. Rev Esp Cir Ortop Traumatol [Internet]. 2023;67(5):426–45. Available from: http://dx.doi.org/10.1016/j.recot.2023.04.010 | Overview not focused on effects of health interventions |
| Salehi AM, Jenabi E, Farashi S, Aghababaei S, Salimi Z. The environmental risk factors related to uterine leiomyoma: An umbrella review. J Gynecol Obstet Hum Reprod [Internet]. 2023;52(1):102517. Available from: https://www.ncbi.nlm.nih.gov/pubmed/36481492 | Overview not focused on effects of health interventions |
| Salih S, O’Callaghan J, Salih M, Walker J, Magar RR, Knight S, et al. Trends in systematic reviews of kidney transplantation: A 10-year analysis of the evidence base. Transplant Rev [Internet]. 2023;37(2):100759. Available from: http://dx.doi.org/10.1016/j.trre.2023.100759 | Overview not focused on effects of health interventions |
| Schwingshackl L, Stadelmaier J, Lay R, Griebler U, De Santis KK, Zeeb H, et al. In Cochrane nutrition reviews assessment of dietary adherence varied considerably. J Clin Epidemiol [Internet]. 2023;158:1–9. Available from: https://www.ncbi.nlm.nih.gov/pubmed/36965600 | Overview not focused on effects of health interventions |
| SeyedAlinaghi S, Afsahi AM, Shahidi R, Kianzad S, Pashaei Z, Mirahmad M, et al. Effects of Smoking on COVID-19 Management and Mortality: An Umbrella Review. J Smok Cessat [Internet]. 2023;2023:7656135. Available from: http://dx.doi.org/10.1155/2023/7656135 | Overview not focused on effects of health interventions |
| SeyedAlinaghi S, Bagheri A, Razi A, Mojdeganlou P, Mojdeganlou H, Afsahi AM, et al. Late Complications of COVID-19; An Umbrella Review on Current Systematic Reviews. Arch Acad Emerg Med [Internet]. 2023;11(1):e28. Available from: http://dx.doi.org/10.22037/aaem.v11i1.1907 | Overview not focused on effects of health interventions |
| Shahabi S, Mojgani P, Lankarani KB, Jalali M. The quality of systematic reviews/meta-analyses assessing the effects of ankle-foot orthosis on clinical outcomes in stroke patients: A methodological systematic review. Health Sci Rep [Internet]. 2023;6(3):e1130. Available from: http://dx.doi.org/10.1002/hsr2.1130 | Overview not focused on effects of health interventions |
| Shang Z, Wanyan P, Zhang B, Wang M, Wang X. A systematic review, umbrella review, and quality assessment on clinical translation of stem cell therapy for knee osteoarthritis: Are we there yet? Stem Cell Res Ther [Internet]. 2023;14(1):91. Available from: http://dx.doi.org/10.1186/s13287-023-03332-5 | Not an overview |
| Siebert M, Caquelin L, Madera M, Acosta-Dighero R, Naudet F, Roqué M. Assessing the magnitude of changes from protocol to publication-a survey on Cochrane and non-Cochrane Systematic Reviews. PeerJ [Internet]. 2023;11:e16016. Available from: http://dx.doi.org/10.7717/peerj.16016 | Overview not focused on effects of health interventions |
| Silva FM, Lima J, Teixeira PP, Grezzana GB, Figueiro M, Colombo T, et al. Risk of bias and certainty of evidence on the association between obesity and mortality in patients with SARS-COV-2: An umbrella review of meta-analyses. Clin Nutr ESPEN [Internet]. 2023;53:13–25. Available from: http://dx.doi.org/10.1016/j.clnesp.2022.08.014 | Overview not focused on effects of health interventions |
| Silva HECD, Santos GNM, Leite AF, Mesquita CRM, Figueiredo PTS, Stefani CM, et al. The use of artificial intelligence tools in cancer detection compared to the traditional diagnostic imaging methods: An overview of the systematic reviews. PLoS One [Internet]. 2023;18(10):e0292063. Available from: http://dx.doi.org/10.1371/journal.pone.0292063 | Overview not focused on effects of health interventions |
| Soorojebally Y, Neuzillet Y, Lebret T, Allory Y, Descotes F, Ferlicot S, et al. Photodynamic cystoscopy for bladder cancer diagnosis and for NMIBC follow-up: An overview of systematic reviews and meta-analyses. Prog Urol [Internet]. 2023;33(6):307–18. Available from: https://www.ncbi.nlm.nih.gov/pubmed/37088584 | Overview not focused on effects of health interventions |
| Soriano VX, Ciciulla D, Gell G, Wang Y, Peters RL, McWilliam V, et al. Complementary and Allergenic Food Introduction in Infants: An Umbrella Review. Pediatrics [Internet]. 2023;151(2). Available from: https://www.ncbi.nlm.nih.gov/pubmed/36704902 | Overview not focused on effects of health interventions |
| Sowers CB, Carrero AC, Cyrus JW, Ross JA, Golladay GJ, Patel NK. Return to Sports After Total Hip Arthroplasty: An Umbrella Review for Consensus Guidelines. Am J Sports Med [Internet]. 2023;51(1):271–8. Available from: http://dx.doi.org/10.1177/03635465211045698 | Overview not focused on effects of health interventions |
| Soysal P, Veronese N, Ippoliti S, Pizzol D, Carrie AM, Stefanescu S, et al. The impact of urinary incontinence on multiple health outcomes: an umbrella review of meta-analysis of observational studies. Aging Clin Exp Res [Internet]. 2023;35(3):479–95. Available from: https://www.ncbi.nlm.nih.gov/pubmed/36637774 | Overview not focused on effects of health interventions |
| Stanley TD, Ioannidis JPA, Maier M, Doucouliagos H, Otte WM, Bartoš F. Unrestricted weighted least squares represent medical research better than random effects in 67,308 Cochrane meta-analyses. J Clin Epidemiol [Internet]. 2023;157:53–8. Available from: https://www.ncbi.nlm.nih.gov/pubmed/36889450 | Overview not focused on effects of health interventions |
| Stearns JA, Avedzi HM, Yim D, Spence JC, Labbaf F, Lamboglia CG, et al. An Umbrella Review of the Best and Most Up-to-Date Evidence on the Built Environment and Physical Activity in Older Adults ≥60 Years. Public Health Rev [Internet]. 2023;44:1605474. Available from: http://dx.doi.org/10.3389/phrs.2023.1605474 | Overview not focused on effects of health interventions |
| Stockman D, Haney L, Uzieblo K, Littleton H, Keygnaert I, Lemmens G, et al. An ecological approach to understanding the impact of sexual violence: a systematic meta-review. Front Psychol [Internet]. 2023;14:1032408. Available from: http://dx.doi.org/10.3389/fpsyg.2023.1032408 | Overview not focused on effects of health interventions |
| Subramaniam P, Thillainathan P, Mat Ghani NA, Sharma S. Life Story Book to enhance communication in persons with dementia: A systematic review of reviews. PLoS One [Internet]. 2023;18(10):e0291620. Available from: http://dx.doi.org/10.1371/journal.pone.0291620 | Not an overview |
| Taiwo BO, Romdhani H, Lafeuille MH, Bhojwani R, Milbers K, Donga P. Treatment and comorbidity burden among people living with HIV: a review of systematic literature reviews. J Drug Assess [Internet]. 2023;12(1):1–11. Available from: http://dx.doi.org/10.1080/21556660.2022.2149963 | Overview not focused on effects of health interventions |
| Tan EJ, Raut T, Le LK, Hay P, Ananthapavan J, Lee YY, et al. The association between eating disorders and mental health: an umbrella review. J Eat Disord [Internet]. 2023;11(1):51. Available from: http://dx.doi.org/10.1186/s40337-022-00725-4 | Overview not focused on effects of health interventions |
| Tannou T, Lihoreau T, Couture M, Giroux S, Wang RH, Spalla G, et al. Is research on “smart living environments” based on unobtrusive technologies for older adults going in circles? Evidence from an umbrella review. Ageing Res Rev [Internet]. 2023;84:101830. Available from: https://www.ncbi.nlm.nih.gov/pubmed/36565962 | Not an overview |
| Tay E, Makeham M, Laba TL, Baysari M. Prescription drug monitoring programs evaluation: A systematic review of reviews. Drug Alcohol Depend [Internet]. 2023;247:109887. Available from: http://dx.doi.org/10.1016/j.drugalcdep.2023.109887 | Overview not focused on effects of health interventions |
| Taylor-Rowan M, Nafisi S, Owen R, Duffy R, Patel A, Burton JK, et al. Informant-based screening tools for dementia: an overview of systematic reviews. Psychol Med [Internet]. 2023;53(2):580–9. Available from: https://www.ncbi.nlm.nih.gov/pubmed/34030753 | Overview not focused on effects of health interventions |
| Tope P, Farah E, Ali R, El-Zein M, Miller WH, Franco EL. The impact of lag time to cancer diagnosis and treatment on clinical outcomes prior to the COVID-19 pandemic: A scoping review of systematic reviews and meta-analyses. Elife [Internet]. 2023;12. Available from: http://dx.doi.org/10.7554/eLife.81354 | Overview not focused on effects of health interventions |
| Travica N, Lotfaliany M, Marriott A, Safavynia SA, Lane MM, Gray L, et al. Peri-Operative Risk Factors Associated with Post-Operative Cognitive Dysfunction (POCD): An Umbrella Review of Meta-Analyses of Observational Studies. J Clin Med Res [Internet]. 2023;12(4). Available from: http://dx.doi.org/10.3390/jcm12041610 | Overview not focused on effects of health interventions |
| Travis N, Knoll M, Cook S, Oh H, Cadham CJ, Sánchez-Romero LM, et al. Chemical Profiles and Toxicity of Electronic Cigarettes: An Umbrella Review and Methodological Considerations. Int J Environ Res Public Health [Internet]. 2023;20(3). Available from: http://dx.doi.org/10.3390/ijerph20031908 | Overview not focused on effects of health interventions |
| Trimboli P, Mian C, Piccardo A, Treglia G. Diagnostic tests for medullary thyroid carcinoma: an umbrella review. Endocrine [Internet]. 2023;81(2):183–93. Available from: http://dx.doi.org/10.1007/s12020-023-03326-6 | Overview not focused on effects of health interventions |
| Tsutsumi Y, Tsujimoto Y, Tajika A, Omae K, Fujii T, Onishi A, et al. Proportion attributable to contextual effects in general medicine: a meta-epidemiological study based on Cochrane reviews. BMJ Evid Based Med [Internet]. 2023;28(1):40–7. Available from: http://dx.doi.org/10.1136/bmjebm-2021-111861 | Overview not focused on effects of health interventions |
| van Dongen A, Stewart D, Garry J, McCambridge J. Measurement of person-centred consultation skills among healthcare practitioners: a systematic review of reviews of validation studies. BMC Med Educ [Internet]. 2023;23(1):211. Available from: http://dx.doi.org/10.1186/s12909-023-04184-6 | Overview not focused on effects of health interventions |
| Veronese N, Honvo G, Bruyère O, Rizzoli R, Barbagallo M, Maggi S, et al. Knee osteoarthritis and adverse health outcomes: an umbrella review of meta-analyses of observational studies. Aging Clin Exp Res [Internet]. 2023;35(2):245–52. Available from: http://dx.doi.org/10.1007/s40520-022-02289-4 | Overview not focused on effects of health interventions |
| Vivekanantha P, Shah A, Hoit G, Ayeni O, Whelan D. Predictors of Increased Fragility Index Scores in Surgical Randomized Controlled Trials: An Umbrella Review. World J Surg [Internet]. 2023;47(5):1163–73. Available from: https://www.ncbi.nlm.nih.gov/pubmed/36719446 | Overview not focused on effects of health interventions |
| Voss T, Krag M, Martiny F, Heleno B, Jørgensen KJ, Brandt Brodersen J. Quantification of overdiagnosis in randomised trials of cancer screening: an overview and re-analysis of systematic reviews. Cancer Epidemiol [Internet]. 2023;84:102352. Available from: http://dx.doi.org/10.1016/j.canep.2023.102352 | Overview not focused on effects of health interventions |
| Waddell A, Kunstler B, Lennox A, Pattuwage L, Grundy EAC, Tsering D, et al. How effective are interventions in optimizing workplace mental health and well-being? A scoping review of reviews and evidence map. Scand J Work Environ Health [Internet]. 2023;49(4):235–48. Available from: http://dx.doi.org/10.5271/sjweh.4087 | Not an overview |
| Wang P, Chen B, Huang Y, Li J, Cao D, Chen Z, et al. Selenium intake and multiple health-related outcomes: an umbrella review of meta-analyses. Front Nutr [Internet]. 2023;10:1263853. Available from: http://dx.doi.org/10.3389/fnut.2023.1263853 | Does not include an explicit list of the included reviews |
| Wang P, Giovannucci EL. Are exposure-disease relationships assessed in cohorts of health professionals generalizable?: a comparative analysis based on WCRF/AICR systematic literature reviews. Cancer Causes Control [Internet]. 2023;34(1):39–45. Available from: https://www.ncbi.nlm.nih.gov/pubmed/36197566 | Not an overview |
| Wang T, Tan JB, Liu XL, Zhao I. Barriers and enablers to implementing clinical practice guidelines in primary care: an overview of systematic reviews. BMJ Open [Internet]. 2023;13(1):e062158. Available from: http://dx.doi.org/10.1136/bmjopen-2022-062158 | Overview not focused on effects of health interventions |
| Wang Y, Wen N, Xiong X, Li B, Lu J. Biliary drainage in malignant biliary obstruction: an umbrella review of randomized controlled trials. Front Oncol [Internet]. 2023;13:1235490. Available from: http://dx.doi.org/10.3389/fonc.2023.1235490 | Not an overview |
| Witteveen AB, Young SY, Cuijpers P, Ayuso-Mateos JL, Barbui C, Bertolini F, et al. COVID-19 and common mental health symptoms in the early phase of the pandemic: An umbrella review of the evidence. PLoS Med [Internet]. 2023;20(4):e1004206. Available from: http://dx.doi.org/10.1371/journal.pmed.1004206 | Overview not focused on effects of health interventions |
| Wohlfart O, Wagner I. Teachers’ role in digitalizing education: an umbrella review. Educ Technol Res Dev [Internet]. 2023;71(2):339–65. Available from: http://dx.doi.org/10.1007/s11423-022-10166-0 | Not an overview |
| Wu J, Zhang H, Shao J, Chen D, Xue E, Huang S, et al. Healthcare for Older Adults with Multimorbidity: A Scoping Review of Reviews. Clin Interv Aging [Internet]. 2023;18:1723–35. Available from: http://dx.doi.org/10.2147/CIA.S425576 | Not an overview |
| Wu TT, Zou YL, Xu KD, Jiang XR, Zhou MM, Zhang SB, et al. Insomnia and multiple health outcomes: umbrella review of meta-analyses of prospective cohort studies. Public Health [Internet]. 2023;215:66–74. Available from: https://www.ncbi.nlm.nih.gov/pubmed/36645961 | Overview not focused on effects of health interventions |
| Wyngaert KV, Nédée ML, Piessevaux O, De Martelaer T, Van Biesen W, Cocquyt V, et al. The role and the composition of a liaison team to facilitate the transition of adolescents and young adults: an umbrella review. Eur J Pediatr [Internet]. 2023;182(4):1483–94. Available from: https://www.ncbi.nlm.nih.gov/pubmed/36735061 | Overview not focused on effects of health interventions |
| Xie Y, Xu J, Zhou D, Guo M, Zhang M, Gao Y, et al. Micronutrient perspective on COVID-19: Umbrella review and reanalysis of meta-analyses. Crit Rev Food Sci Nutr [Internet]. 2023;1–19. Available from: https://www.ncbi.nlm.nih.gov/pubmed/36794398 | Not an overview |
| Xuan C, Zhang B, Jia X. The Effect of Human Settlement Pedestrian Environment on Gait of Older People: An Umbrella Review. Int J Environ Res Public Health [Internet]. 2023;20(2). Available from: http://dx.doi.org/10.3390/ijerph20021567 | Overview not focused on effects of health interventions |
| Ying G, Zhao G, Xu X, Su S, Xie X. Association of age-related hearing loss with cognitive impairment and dementia: an umbrella review. Front Aging Neurosci [Internet]. 2023;15:1241224. Available from: http://dx.doi.org/10.3389/fnagi.2023.1241224 | Overview not focused on effects of health interventions |
| Ying ZQ, Li DL, Zheng XY, Zhang XF, Pan CW. Risk factors for myopia among children and adolescents: an umbrella review of published meta-analyses and systematic reviews. Br J Ophthalmol [Internet]. 2023; Available from: https://www.ncbi.nlm.nih.gov/pubmed/36754586 | Overview not focused on effects of health interventions |
| Yu B, Ma SQ, Huang HP, Zhong Z, Yu S, Huang K, et al. Research methods and efficacy of acupuncture in the treatment of Parkinson’s disease: a scoping review of systematic reviews and meta-analyses. Front Neurol [Internet]. 2023;14:1196446. Available from: http://dx.doi.org/10.3389/fneur.2023.1196446 | Overview not focused on effects of health interventions |
| Zarzar AM, Sales PHDH, Barros AWP, Arreguy IMS, Carvalho AAT, Leão JC. Effectiveness of dental implants in patients undergoing radiotherapy for head and neck cancer: An umbrella review. Spec Care Dentist [Internet]. 2023; Available from: https://onlinelibrary.wiley.com/doi/10.1111/scd.12840 | Overview not focused on effects of health interventions |
| Zavalis EA, Rameau A, Saraswathula A, Vist J, Schuit E, Ioannidis JPA. Availability of evidence and comparative effectiveness for surgical versus drug interventions: an overview of systematic reviews. medRxiv [Internet]. 2023; Available from: http://dx.doi.org/10.1101/2023.01.30.23285207 | Preprint |
| Zeng L, Yao L, Wang Y, Han MA, Granholm A, Nampo F, et al. Presentation approaches for enhancing interpretability of patient-reported outcomes in meta-analyses: a systematic survey of Cochrane reviews. J Clin Epidemiol [Internet]. 2023;158:119–26. Available from: http://dx.doi.org/10.1016/j.jclinepi.2023.03.027 | Overview not focused on effects of health interventions |
| Zha B, Luo Y, Kamili M, Zha X. Non-coding RNAs and gastrointestinal cancers prognosis: an umbrella review of systematic reviews and meta-analyses of observational studies. Front Oncol [Internet]. 2023;13:1193665. Available from: http://dx.doi.org/10.3389/fonc.2023.1193665 | Overview not focused on effects of health interventions |
| Zhang Y, Leuk JS, Teo WP. Domains, Feasibility, Effectiveness, Cost, and Acceptability of Telehealth in Aging Care: Scoping Review of Systematic Reviews. JMIR Aging [Internet]. 2023;6:e40460. Available from: http://dx.doi.org/10.2196/40460 | Not an overview |
| Zhang Y, Liu N, Li Y, Long Y, Baumgartner J, Adamkiewicz G, et al. Neighborhood infrastructure-related risk factors and non-communicable diseases: a systematic meta-review. Environ Health [Internet]. 2023;22(1):2. Available from: http://dx.doi.org/10.1186/s12940-022-00955-8 | Overview not focused on effects of health interventions |
| Zhang Y, Zang Y, Ren J, Guo W, Disantis A, Liu S, et al. Use of Patient-Reported Outcome Measures in Lower Extremity Research. Int J Sports Phys Ther [Internet]. 2023;V18(3):645–52. Available from: http://dx.doi.org/10.26603/001c.74698 | Overview not focused on effects of health interventions |
| Zhao H, Wang M, Peng X, Zhong L, Liu X, Shi Y, et al. Fish consumption in multiple health outcomes: an umbrella review of meta-analyses of observational and clinical studies. Ann Transl Med [Internet]. 2023;11(3):152. Available from: http://dx.doi.org/10.21037/atm-22-6515 | Overview not focused on effects of health interventions |
| Ziebart C, Bobos P, Furtado R, Dabbagh A, MacDermid J. Patient-reported outcome measures used for hand and wrist disorders: An overview of systematic reviews. J Hand Ther [Internet]. 2023;36(3):719–29. Available from: https://www.ncbi.nlm.nih.gov/pubmed/36914499 | Overview not focused on effects of health interventions |

##

## Appendix 4. List of overviews meeting eligibility criteria and their main characteristics.

| **Study ID** | **PMID** | **Title** | **Scope** | | | **Total SRs included** | **Reporting guideline used^1^** | **Overlap addressing strategy** |
| --- | --- | --- | --- | --- | --- | --- | --- | --- |
|  |  |  | **Population** | **Interventions** | **Overall** |  |  |  |
| Adams 2023 | 37024777 | Effectiveness and implementation of interventions for health promotion in urgent and emergency care settings: an umbrella review. | Broad | Broad | Broad | 18 | PRISMA | No mention to overlap |
| Ambagtsheer 2023 | 37088103 | Does CGA Improve Health Outcomes in the Community? An Umbrella Review. | Broad | Broad | Broad | 11 | PRISMA | In the 'Synthesis and presentation and summary of findings' step: Visual representation of overlap other than matrices of evidence |
| Amiri Khosroshahi 2023 | 37072129 | Effect of probiotic supplementation on chemotherapy- and radiotherapy-related diarrhoea in patients with cancer: an umbrella review of systematic reviews and meta-analyses. | Narrow | Narrow | Narrow | 13 | PRIOR | No mention to overlap |
| Angoorani 2023 | 37359018 | The effects of probiotics, prebiotics, and synbiotics on polycystic ovarian syndrome: an overview of systematic reviews. | Narrow | Broad | Broad | 8 | Not reported | No mention to overlap |
| Ardila 2023 | 37435111 | Clinical Efficacy of Platelet Derivatives in Periodontal Tissue Regeneration: An Umbrella Review. | Narrow | Narrow | Narrow | 8 | PRISMA | In the 'Synthesis and presentation and summary of findings' step: Visual representation of overlap through matrices of evidence |
| Arribas-Pascual 2023 | 36769437 | Effects of Physiotherapy on Pain and Mouth Opening in Temporomandibular Disorders: An Umbrella and Mapping Systematic Review with Meta-Meta-Analysis. | Narrow | Broad | Broad | 31 | PRIOR | In the 'Synthesis and presentation and summary of findings' step: Quantifying overlap with CCA  In the 'Synthesis and presentation and summary of findings' step: Visual representation of overlap through matrices of evidence |
| Barbosa 2023 | 36894478 | Rapid maxillary expansion in pediatric patients with obstructive sleep apnea: an umbrella review. | Narrow | Narrow | Narrow | 7 | Not reported | No mention to overlap |
| Barrett 2023 | 36789663 | Interventions to Reduce Parental Substance Use, Domestic Violence and Mental Health Problems, and Their Impacts Upon Children's Well-Being: A Systematic Review of Reviews and Evidence Mapping. | Broad | Broad | Broad | 62 | Not reported | In the 'Data extraction / Synthesis and presentation and summary of findings' step: Extracting and analysing primary study data |
| Bellon 2023 | 36871416 | Effectiveness of orthopaedic treatments on the enlargement of the upper airways: Overview of systematic reviews. | Narrow | Narrow | Narrow | 10 | PRISMA | No mention to overlap |
| Belloni 2023 | 36973125 | Non-pharmacologic interventions for improving cancer-related fatigue (CRF): A systematic review of systematic reviews and pooled meta-analysis. | Narrow | Broad | Broad | 28 | PRISMA | In the 'Synthesis and presentation and summary of findings' step: Quantifying overlap with a method different than CCA  In the 'Synthesis and presentation and summary of findings' step: Statistical methods (e.g., sensitivity analysis) |
| Belloni 2023 | 36566113 | A Systematic Review of Systematic Reviews and a Pooled Meta-Analysis on Complementary and Integrative Medicine for Improving Cancer-Related Fatigue. | Narrow | Broad | Broad | 22 | PRISMA | In the 'Synthesis and presentation and summary of findings' step: Quantifying overlap with a method different than CCA |
| Beygi 2023 | 37383966 | An Overview of Reviews on the Effects of Acceptance and Commitment Therapy (ACT) on Depression and Anxiety. | Narrow | Narrow | Narrow | 25 | Not reported | No mention to overlap |
| Boccia 2023 | 36671315 | Local and Systemic Antibiotics in Peri-Implantitis Management: An Umbrella Review | Narrow | Narrow | Narrow | 7 | PRISMA | No mention to overlap |
| Bonczar 2023 | 36584870 | A complete analysis of the surgical treatment for cubital tunnel syndrome: an umbrella review. | Narrow | Narrow | Narrow | 14 | PRISMA | No mention to overlap |
| Bonnechère 2023 | 36920851 | Cognitive Computerized Training for Older Adults and Patients with Neurological Disorders: Do the Amount and Training Modality Count? An Umbrella Meta-Regression Analysis. | Broad | Narrow | Broad | 8 | PRISMA | No mention to overlap |
| Botwright 2023 | 36823618 | Which interventions for alcohol use should be included in a universal healthcare benefit package? An umbrella review of targeted interventions to address harmful drinking and dependence. | Narrow | Broad | Broad | 86 | PRIOR | In the 'Data extraction / Synthesis and presentation and summary of findings' step: Extracting and analysing primary study data |
| Bracchiglione 2023 | 36765723 | Systemic Oncological Treatments versus Supportive Care for Patients with Advanced Hepatobiliary Cancers: An Overview of Systematic Reviews. | Narrow | Broad | Broad | 18 | PRIOR | In the 'Synthesis and presentation and summary of findings' step: Quantifying overlap with CCA  In the 'Synthesis and presentation and summary of findings' step: Visual representation of overlap through matrices of evidence  In the 'Data extraction / Synthesis and presentation and summary of findings' step: Extracting and analysing primary study data |
| Brini 2023 | 36587461 | Efficacy and safety of transcranial magnetic stimulation for treating major depressive disorder: An umbrella review and re-analysis of published meta-analyses of randomised controlled trials. | Narrow | Broad | Broad | 29 | Not reported | No mention to overlap |
| Brown 2023 | 36828754 | Non-pharmacological interventions for self-management of fatigue in adults: An umbrella review of potential interventions to support patients recovering from critical illness. | Broad | Broad | Broad | 10 | PRISMA | No mention to overlap |
| Brusola 2023 | 36806522 | Effectiveness of physical therapy interventions on post-stroke spasticity: An umbrella review. | Narrow | Broad | Broad | 8 | Not reported | No mention to overlap |
| Cardle 2023 | 36879651 | Mindfulness and Chronic Musculoskeletal Pain: An Umbrella Review. | Broad | Broad | Broad | 19 | PRISMA | In the 'Synthesis and presentation and summary of findings' step: Quantifying overlap with a method different than CCA |
| Cashin 2023 | 37014979 | Pharmacological treatments for low back pain in adults: an overview of Cochrane Reviews. | Narrow | Broad | Broad | 7 | Not reported | In the 'Synthesis and presentation and summary of findings' step: Quantifying overlap with CCA |
| Cedenilla Ramón 2023 | 36975439 | Psychosocial Interventions for the Treatment of Cancer-Related Fatigue: An Umbrella Review. | Narrow | Broad | Broad | 11 | Not reported | No mention to overlap |
| Chan 2023 | 34786652 | Effectiveness and implementation of models of cancer survivorship care: an overview of systematic reviews. | Narrow | Broad | Broad | 12 | PRISMA | In the 'Synthesis and presentation and summary of findings' step: Quantifying overlap with CCA  In the 'Synthesis and presentation and summary of findings' step: Visual representation of overlap through matrices of evidence  In the 'Synthesis and presentation and summary of findings' step: Select reviews |
| Chen 2023 | 36814496 | Function of sildenafil on diseases other than urogenital system: An umbrella review. | Broad | Narrow | Broad | 77 | PRISMA | No mention to overlap |
| Chys 2023 | 36769852 | Clinical Effectiveness of Dry Needling in Patients with Musculoskeletal Pain-An Umbrella Review. | Broad | Narrow | Broad | 36 | PRISMA | In the 'Synthesis and presentation and summary of findings' step: Quantifying overlap with CCA  In the 'Synthesis and presentation and summary of findings' step: Visual representation of overlap through matrices of evidence  In the 'Synthesis and presentation and summary of findings' step: Select reviews |
| Ciria 2023 | 36973359 | An umbrella review of randomized control trials on the effects of physical exercise on cognition. | Broad | Broad | Broad | 24 | PRISMA | In the 'Synthesis and presentation and summary of findings' step: Quantifying overlap with a method different than CCA  In the 'Synthesis and presentation and summary of findings' step: Visual representation of overlap other than matrices of evidence |
| Contillo 2023 | 37105550 | Exercise and Protein Supplementation Recommendations for Older Adults With Sarcopenic Obesity: A Meta-Review. | Narrow | Broad | Broad | 5 | PRISMA | In the 'Synthesis and presentation and summary of findings' step: Quantifying overlap with a method different than CCA  In the 'Synthesis and presentation and summary of findings' step: Visual representation of overlap other than matrices of evidence |
| Croatto 2023 | 36138129 | The impact of pharmacological and non-pharmacological interventions on physical health outcomes in people with mood disorders across the lifespan: An umbrella review of the evidence from randomised controlled trials. | Broad | Broad | Broad | 97 | Not reported | No mention to overlap |
| Dabbaghi Varnousfaderani 2023 | 37614320 | Alleviating effects of coenzyme Q10 supplements on biomarkers of inflammation and oxidative stress: results from an umbrella meta-analysis. | Broad | Narrow | Broad | 13 | PRISMA | Addressed only in the discussion section |
| de Alvarenga 2023 | 36760221 | What has been done to improve learning for intellectual disability? An umbrella review of published meta-analyses and systematic reviews. | Narrow | Broad | Broad | 59 | PRISMA | No mention to overlap |
| de-la-Casa-Almeida 2023 | 37115606 | Mind-body exercises for osteoarthritis: an overview of systematic reviews including 32 meta-analyses. | Narrow | Narrow | Narrow | 32 | PRIOR | In the 'Synthesis and presentation and summary of findings' step: Quantifying overlap with CCA  In the 'Synthesis and presentation and summary of findings' step: Visual representation of overlap other than matrices of evidence |
| Denova-Gutiérrez 2023 | 36771481 | Overview of Systematic Reviews of Health Interventions for the Prevention and Treatment of Overweight and Obesity in Children. | Narrow | Broad | Broad | 10 | PRISMA | In the 'Synthesis and presentation and summary of findings' step: Quantifying overlap with CCA  In the 'Synthesis and presentation and summary of findings' step: Visual representation of overlap other than matrices of evidence |
| Desaunay 2023 | 36853497 | Benefits and Risks of Antidepressant Drugs During Pregnancy: A Systematic Review of Meta-analyses. | Narrow | Narrow | Narrow | 51 | PRISMA | In the 'Synthesis and presentation and summary of findings' step: Quantifying overlap with CCA |
| Dhingra 2023 | 36562900 | Tranexamic acid in emergency medicine. An overview of reviews. | Narrow | Narrow | Narrow | 13 | PRIOR | In the 'Synthesis and presentation and summary of findings' step: Quantifying overlap with CCA |
| Di Domenico 2023 | 36261746 | Effectiveness of periodontal treatment to improve glycemic control: an umbrella review. | Narrow | Narrow | Narrow | 16 | PRISMA | No mention to overlap |
| Di Mario 2023 | 36074961 | The Use of Yoga and Mindfulness-based Interventions to Reduce Stress and Burnout in Healthcare Workers: An Umbrella Review. | Narrow | Narrow | Narrow | 12 | PRISMA | No mention to overlap |
| Di Spirito 2023 | 36826180 | Periodontal Management in Periodontally Healthy Orthodontic Patients with Fixed Appliances: An Umbrella Review of Self-Care Instructions and Evidence-Based Recommendations. | Narrow | Narrow | Narrow | 17 | PRISMA | No mention to overlap |
| Dong 2023 | 37720380 | Effect of probiotics intake on constipation in children: an umbrella review. | Narrow | Narrow | Narrow | 9 | Not reported | No mention to overlap |
| Dörfler 2023 | 36779370 | Umbrella review: Summary of findings for acupuncture as treatment for radiation-induced xerostomia. | Narrow | Narrow | Narrow | 8 | Not reported | In the 'Synthesis and presentation and summary of findings' step: Visual representation of overlap through matrices of evidence |
| Drovandi 2023 | 34008448 | Remotely Delivered Monitoring and Management of Diabetes-Related Foot Disease: An Overview of Systematic Reviews. | Narrow | Broad | Broad | 8 | PRISMA | In the 'Synthesis and presentation and summary of findings' step: Visual representation of overlap other than matrices of evidence |
| Edbrooke 2023 | 36902659 | Exercise across the Lung Cancer Care Continuum: An Overview of Systematic Reviews. | Narrow | Narrow | Narrow | 30 | PRISMA | In the 'Synthesis and presentation and summary of findings' step: Quantifying overlap with CCA  In the 'Synthesis and presentation and summary of findings' step: Visual representation of overlap through matrices of evidence  In the 'Synthesis and presentation and summary of findings' step: Select reviews |
| Els 2023 | 36961252 | High-dose opioids for chronic non-cancer pain: an overview of Cochrane Reviews. | Broad | Narrow | Broad | 0 | Not reported | No mention to overlap |
| Eltaybani 2023 | 37060618 | Effectiveness of home visit nursing on improving mortality, hospitalization, institutionalization, satisfaction, and quality of life among older people: Umbrella review. | Broad | Broad | Broad | 10 | Not reported | In the 'Synthesis and presentation and summary of findings' step: Quantifying overlap with CCA  In the 'Synthesis and presentation and summary of findings' step: Visual representation of overlap through matrices of evidence |
| Fadel 2023 | 36941932 | Interventions on Well-being, Occupational Health, and Aging of Healthcare Workers: A Scoping Review of Systematic Reviews. | Narrow | Broad | Broad | 13 | PRISMA | No mention to overlap |
| Fan 2023 | 37113506 | Efficacy of Acupuncture in the Treatment of Essential Hypertension: An Overview of Systematic Reviews and Meta-Analyses. | Narrow | Narrow | Narrow | 14 | PRISMA | No mention to overlap |
| Fastner 2023 | 37099847 | Skin assessments and interventions for maintaining skin integrity in nursing practice: An umbrella review. | Broad | Broad | Broad | 12 | Not reported | Addressed only in the discussion section |
| Fausto 2023 | 36696648 | An umbrella systematic review of the effect of physical exercise on mental health of women in menopause. | Narrow | Broad | Broad | 9 | PRISMA | In the 'Synthesis and presentation and summary of findings' step: Quantifying overlap with a method different than CCA  In the 'Synthesis and presentation and summary of findings' step: Visual representation of overlap other than matrices of evidence |
| Feng 2023 | 36908468 | Effectiveness and safety of manual therapy for knee osteoarthritis: An overview of systematic reviews and meta-analyses. | Narrow | Broad | Broad | 11 | Not reported | In the 'Data extraction / Synthesis and presentation and summary of findings' step: Extracting and analysing primary study data |
| Ferreira 2023 | 36725015 | Efficacy, safety, and tolerability of antidepressants for pain in adults: overview of systematic reviews. | Broad | Narrow | Broad | 26 | PRIOR | In the 'Synthesis and presentation and summary of findings' step: Quantifying overlap with CCA  In the 'Synthesis and presentation and summary of findings' step: Visual representation of overlap through matrices of evidence  In the 'Synthesis and presentation and summary of findings' step: Select reviews  In the 'Synthesis and presentation and summary of findings' step: Statistical methods (e.g., sensitivity analysis) |
| Ferreira 2023 | 36402001 | The effect of different exercise programs on sarcopenia criteria in older people: A systematic review of systematic reviews with meta-analysis. | Narrow | Broad | Broad | 5 | PRISMA | No mention to overlap |
| Fieiras 2023 | 35101925 | Risperidone and aripiprazole for autism spectrum disorder in children: an overview of systematic reviews. | Narrow | Narrow | Narrow | 6 | PRISMA | In the 'eligibility criteria' step: Include all reviews |
| Franquez 2023 | 36758047 | Interventions for depression and anxiety among people with diabetes mellitus: Review of systematic reviews. | Narrow | Broad | Broad | 13 | PRISMA | Addressed only in the discussion section |
| Gao 2023 | 37282973 | [Overview of systematic reviews of Chinese herbal injections for sepsis]. | Narrow | Narrow | Narrow | 27 | Not reported | No mention to overlap |
| Gao 2023 | 37006488 | Repetitive transcranial magnetic stimulation for post-stroke depression: An overview of systematic reviews. | Narrow | Narrow | Narrow | 13 | Not reported | No mention to overlap |
| Gazzaniga 2023 | 36872367 | Effect of digoxin on all-cause and cardiovascular mortality in patients with atrial fibrillation with and without heart failure: an umbrella review of systematic reviews and 12 meta-analyses. | Narrow | Narrow | Narrow | 11 | PRISMA | No mention to overlap |
| Gianfredi 2023 | 36240456 | Association between dietary patterns and depression: an umbrella review of meta-analyses of observational studies and intervention trials. | Broad | Broad | Broad | 19 | PRISMA | Addressed only in the discussion section |
| Grillich 2023 | 36893335 | The effectiveness of interventions to prevent loneliness and social isolation in the community-dwelling and old population: an overview of systematic reviews and meta-analysis. | Broad | Broad | Broad | 8 | PRISMA | No mention to overlap |
| Hashemi 2023 | 37059449 | Tooth Graft: An Umbrella Overview. | Narrow | Narrow | Narrow | 9 | PRISMA | In the 'eligibility criteria' step: Exclude reviews without unique primary studies  In the 'Synthesis and presentation and summary of findings' step: Visual representation of overlap through matrices of evidence |
| He 2023 | 37324817 | Effect of family-centered interventions for perinatal depression: an overview of systematic reviews. | Narrow | Broad | Broad | 8 | PRIO-harms | In the 'Synthesis and presentation and summary of findings' step: Quantifying overlap with a method different than CCA  In the 'Synthesis and presentation and summary of findings' step: Quantifying overlap with CCA |
| Hou 2023 | 36781549 | Is physical activity effective against cancer-related fatigue in lung cancer patients? An umbrella review of systematic reviews and meta-analyses. | Narrow | Broad | Broad | 7 | PRISMA | No mention to overlap |
| Jaff 2023 | 36849891 | The effect of selenium therapy in critically ill patients: an umbrella review of systematic reviews and meta-analysis of randomized controlled trials. | Narrow | Narrow | Narrow | 17 | PRIOR | No mention to overlap |
| Janiri 2023 | 36781741 | Lithium use in childhood and adolescence, peripartum, and old age: an umbrella review. | Broad | Narrow | Broad | 20 | PRISMA | In the 'Synthesis and presentation and summary of findings' step: Quantifying overlap with CCA  In the 'Synthesis and presentation and summary of findings' step: Visual representation of overlap through matrices of evidence |
| Jiesisibieke 2023 | 37485047 | Effectiveness and Safety of COVID-19 Vaccinations: An Umbrella Meta-Analysis. | Broad | Narrow | Broad | 19 | PRISMA | No mention to overlap |
| Joo 2023 | 36669435 | Transitional care interventions for supporting frail older adults discharged from hospitals: An umbrella review. | Narrow | Broad | Broad | 9 | PRISMA | No mention to overlap |
| Jun 2023 | 36999074 | Warm needle acupuncture for osteoarthritis: An overview of systematic reviews and meta-analysis. | Narrow | Narrow | Narrow | 15 | PRIOR | In the 'Synthesis and presentation and summary of findings' step: Quantifying overlap with CCA  In the 'Synthesis and presentation and summary of findings' step: Visual representation of overlap through matrices of evidence |
| Khademi 2023 | 37483905 | Effect of premedication on the success of inferior alveolar nerve block in patients diagnosed with irreversible pulpitis: An umbrella review. | Narrow | Narrow | Narrow | 4 | PRISMA | In the 'Synthesis and presentation and summary of findings' step: Select reviews |
| Khan 2023 | 36932766 | Treatment of periodontal disease in pregnancy for the prevention of adverse pregnancy outcomes: a systematic review of systematic reviews. | Narrow | Narrow | Narrow | 17 | PRISMA | No mention to overlap |
| Kip 2023 | 36529109 | Efficacy of psychological interventions for PTSD in distinct populations - An evidence map of meta-analyses using the umbrella review methodology. | Narrow | Broad | Broad | 10 | PRISMA | In the 'eligibility criteria' step: Include all reviews  In the 'Synthesis and presentation and summary of findings' step: Quantifying overlap with a method different than CCA  In the 'Synthesis and presentation and summary of findings' step: Visual representation of overlap other than matrices of evidence |
| Kirvalidze 2023 | 37085312 | Effectiveness of interventions designed to mitigate the negative health outcomes of informal caregiving to older adults: an umbrella review of systematic reviews and meta-analyses. | Narrow | Broad | Broad | 37 | PRIOR | In the 'eligibility criteria step: Select reviews according to pre-specified eligibility criteria or decision rules  In the 'Synthesis and presentation and summary of findings' step: Quantifying overlap with CCA  In the 'Synthesis and presentation and summary of findings' step: Visual representation of overlap through matrices of evidence  In the 'Synthesis and presentation and summary of findings' step: Visual representation of overlap other than matrices of evidence |
| Koh 2023 | 36795479 | Telemedical Interventions for Chronic Obstructive Pulmonary Disease Management: Umbrella Review. | Narrow | Broad | Broad | 7 | Not reported | In the 'eligibility criteria step: Select reviews according to pre-specified eligibility criteria or decision rules |
| Kons 2023 | 36625965 | Effects of Plyometric Training on Physical Performance: An Umbrella Review. | Broad | Narrow | Broad | 29 | PRISMA | No mention to overlap |
| Küçükdeveci 2023 | 36727299 | Overview of Cochrane Systematic Reviews of rehabilitation interventions for persons with rheumatoid arthritis: a mapping synthesis. | Narrow | Broad | Broad | 10 | PRISMA | No mention to overlap |
| Kurdi 2023 | 36205627 | An umbrella review and meta-analysis of renin-angiotensin system drugs use and COVID-19 outcomes. | Narrow | Narrow | Narrow | 47 | PRISMA | In the 'Synthesis and presentation and summary of findings' step: Quantifying overlap with CCA |
| Kwon 2023 | 35973932 | Complementary and integrative medicines for behavioral and psychological symptoms of dementia: Overview of systematic reviews. | Narrow | Broad | Broad | 38 | PRISMA | No mention to overlap |
| Langford 2023 | 36808693 | Patient-targeted interventions for opioid deprescribing: An overview of systematic reviews. | Narrow | Narrow | Narrow | 12 | PRIOR | In the 'eligibility criteria' step: Exclude reviews without unique primary studies  In the 'Data extraction' step: Extract a subset of reviews according to prespecified criteria |
| Lau 2023 | 36919443 | Effects of digital health interventions on the psychological outcomes of perinatal women: umbrella review of systematic reviews and meta-analyses. | Narrow | Broad | Broad | 24 | PRIO-harms | In the 'Synthesis and presentation and summary of findings' step: Quantifying overlap with CCA  In the 'Synthesis and presentation and summary of findings' step: Visual representation of overlap through matrices of evidence  In the 'Synthesis and presentation and summary of findings' step: Statistical methods (e.g., sensitivity analysis) |
| Li 2023 | 37841249 | Impact of concomitant medications on the efficacy of immune checkpoint inhibitors: an umbrella review. | Narrow | Broad | Broad | 23 | PRISMA | In the 'eligibility criteria step: Select reviews according to pre-specified eligibility criteria or decision rules  In the 'Synthesis and presentation and summary of findings' step: Quantifying overlap with CCA  In the 'Synthesis and presentation and summary of findings' step: Visual representation of overlap through matrices of evidence |
| Li 2023 | 37693907 | The efficacy and safety of post-stroke cognitive impairment therapies: an umbrella review. | Narrow | Broad | Broad | 19 | PRISMA | In the 'eligibility criteria step: Select reviews according to pre-specified eligibility criteria or decision rules |
| Li 2023 | 37583426 | An overview of systematic reviews of acupuncture for diabetic gastroparesis. | Narrow | Narrow | Narrow | 10 | Not reported | In the 'Data extraction / Synthesis and presentation and summary of findings' step: Extracting and analysing primary study data |
| Li 2023 | 37515495 | The Role of Complementary and Alternative Medicine on Cancer-Related Fatigue in Adults: An Overview of Systematic Reviews. | Narrow | Broad | Broad | 30 | PRISMA | No mention to overlap |
| Li 2023 | 37465522 | Ginseng and health outcomes: an umbrella review. | Broad | Broad | Broad | 19 | PRISMA | No mention to overlap |
| Li 2023 | 37252111 | Scientific evidence of sodium-glucose cotransporter-2 inhibitors for heart failure with preserved ejection fraction: an umbrella review of systematic reviews and meta-analyses. | Narrow | Narrow | Narrow | 15 | Not reported | In the 'Synthesis and presentation and summary of findings' step: Quantifying overlap with CCA  In the 'Synthesis and presentation and summary of findings' step: Visual representation of overlap through matrices of evidence |
| Li 2023 | 37057304 | Effects of Traditional Chinese Medicine Injections for Anthracyclines-induced Cardiotoxicity: An Overview of Systematic Reviews and Meta-Analyses. | Narrow | Narrow | Narrow | 7 | Not reported | No mention to overlap |
| Li 2023 | 37057067 | Nigella sativa and health outcomes: An overview of systematic reviews and meta-analyses. | Broad | Narrow | Broad | 20 | PRISMA | No mention to overlap |
| Li 2023 | 36999891 | Berberine and health outcomes: An umbrella review. | Broad | Narrow | Broad | 11 | PRISMA | In the 'eligibility criteria step: Select reviews according to pre-specified eligibility criteria or decision rules |
| Li 2023 | 36731639 | Effect of acupuncture in eczema: An overview of systematic reviews. | Narrow | Broad | Broad | 7 | Not reported | No mention to overlap |
| Li 2023 | 36718799 | An umbrella review of the use of platelet-rich plasma in the treatment of androgenetic alopecia. | Narrow | Narrow | Narrow | 28 | Not reported | No mention to overlap |
| Li 2023 | 36701738 | Effectiveness and safety of Inclisiran in hyperlipidemia treatment: An overview of systematic reviews. | Narrow | Narrow | Narrow | 10 | Not reported | In the 'Data extraction' step: Extract a subset of reviews according to prespecified criteria |
| López-Ortiz 2023 | 36342524 | Effects of physical activity and exercise interventions on Alzheimer's disease: an umbrella review of existing meta-analyses. | Narrow | Broad | Broad | 21 | PRISMA | No mention to overlap |
| Lu 2023 | 36863978 | Masking strategy to protect healthcare workers from COVID-19: An umbrella meta-analysis. | Narrow | Narrow | Narrow | 10 | Not reported | No mention to overlap |
| Lu 2023 | 36240975 | Chinese patent medicine Kanglaite injection for non-small-cell lung cancer: An overview of systematic reviews. | Narrow | Narrow | Narrow | 20 | Not reported | In the 'eligibility criteria step: Select reviews according to pre-specified eligibility criteria or decision rules |
| Lugo-Candelas 2023 | 36754341 | Maternal Mental Health and Offspring Brain Development: An Umbrella Review of Prenatal Interventions. | Narrow | Broad | Broad | 30 | PRISMA | No mention to overlap |
| Luo 2023 | 36154604 | Effectiveness and Safety of Immunosuppressive Drug Therapy for Neuromyelitis Optica Spectrum Disorders: An Overview of Meta-Analyses and Systematic Reviews. | Narrow | Narrow | Narrow | 15 | PRISMA | No mention to overlap |
| Maier 2023 | 37228895 | Skill-Mix Changes Targeting Health Promotion and Prevention Interventions and Effects on Outcomes in all Settings (Except Hospitals): Overview of Reviews. | Broad | Broad | Broad | 31 | PRIOR | Addressed only in the discussion section |
| Martínez-Pozas 2023 | 36917046 | Effects of Orthopedic Manual Therapy on Pain Sensitization in Patients With Chronic Musculoskeletal Pain: An Umbrella Review With Meta-Meta-analysis. | Narrow | Broad | Broad | 29 | PRISMA | Not clearly specified |
| Matvienko-Sikar 2023 | 34555958 | Effects of maternal stress and/or anxiety interventions in the first 1000 days:Systematic review of reviews. | Narrow | Broad | Broad | 34 | PRISMA | No mention to overlap |
| Mazzocco 2023 | 36831519 | Evidence for Choosing Qigong as an Integrated Intervention in Cancer Care: An Umbrella Review. | Narrow | Narrow | Narrow | 19 | PRISMA | Not clearly specified |
| McCullock 2023 | 36563518 | The effectiveness of mental illness stigma-reduction interventions: A systematic meta-review of meta-analyses. | Broad | Broad | Broad | 15 | PRISMA | No mention to overlap |
| McDermott 2023 | 37534029 | Digital interventions to moderate physical inactivity and/or nutrition in young people: a Cancer Prevention Europe overview of systematic reviews. | Broad | Broad | Broad | 49 | PRISMA | No mention to overlap |
| McDermott 2023 | 37288171 | Digital interventions to moderate alcohol consumption in young people: a Cancer Prevention Europe overview of systematic reviews. | Narrow | Broad | Broad | 49 | PRISMA | No mention to overlap |
| Meng 2023 | 36039014 | Effectiveness and Safety of Histamine H2 Receptor Antagonists: An Umbrella Review of Meta-Analyses. | Broad | Narrow | Broad | 46 | PRISMA | In the 'Data extraction / Synthesis and presentation and summary of findings' step: Extracting and analysing primary study data |
| Miguel 2023 | 36828633 | Universal, selective and indicated interventions for supporting mental health at the workplace: an umbrella review of meta-analyses. | Narrow | Broad | Broad | 16 | Not reported | In the 'eligibility criteria step: Select reviews according to pre-specified eligibility criteria or decision rules |
| Mitra 2023 | 37039501 | Interventions for patent ductus arteriosus (PDA) in preterm infants: an overview of Cochrane Systematic Reviews. | Narrow | Broad | Broad | 16 | Not reported | No mention to overlap |
| Moslehi 2023 | 36099162 | Effects of nutrition on metabolic and endocrine outcomes in women with polycystic ovary syndrome: an umbrella review of meta-analyses of randomized controlled trials. | Narrow | Broad | Broad | 28 | PRISMA | In the 'eligibility criteria step: Select reviews according to pre-specified eligibility criteria or decision rules |
| Motrico 2023 | 36958130 | Effectiveness of interventions to prevent perinatal depression: An umbrella review of systematic reviews and meta-analysis. | Narrow | Broad | Broad | 19 | PRIOR | In the 'Synthesis and presentation and summary of findings' step: Quantifying overlap with a method different than CCA  In the 'Synthesis and presentation and summary of findings' step: Quantifying overlap with CCA  In the 'Synthesis and presentation and summary of findings' step: Visual representation of overlap other than matrices of evidence |
| Mozafarinia 2023 | 36705274 | An umbrella review of the literature on the effectiveness of goal setting interventions in improving health outcomes in chronic conditions. | Narrow | Broad | Broad | 9 | Not reported | No mention to overlap |
| Musazadeh 2023 | 37727632 | The effect of L-carnitine supplementation on lipid profile in adults: an umbrella meta-analysis on interventional meta-analyses. | Broad | Narrow | Broad | 13 | PRISMA | No mention to overlap |
| Musazadeh 2023 | 37637950 | Remarkable impacts of probiotics supplementation in enhancing of the antioxidant status: results of an umbrella meta-analysis. | Broad | Narrow | Broad | 15 | PRISMA | No mention to overlap |
| Musazadeh 2023 | 36908920 | Effects of synbiotics supplementation on anthropometric and lipid profile parameters: Finding from an umbrella meta-analysis. | Broad | Narrow | Broad | 17 | PRISMA | Addressed only in the discussion section |
| Musazadeh 2023 | 36692292 | Omega-3 polyunsaturated fatty acids in the treatment of non-alcoholic fatty liver disease: An umbrella systematic review and meta-analysis. | Narrow | Narrow | Narrow | 8 | Not reported | Addressed only in the discussion section |
| Musazadeh 2023 | 36509315 | Vitamin D protects against depression: Evidence from an umbrella meta-analysis on interventional and observational meta-analyses. | Broad | Narrow | Broad | 14 | Not reported | No mention to overlap |
| Musazadeh 2023 | 35348020 | Probiotics as an effective therapeutic approach in alleviating depression symptoms: an umbrella meta-analysis. | Broad | Narrow | Broad | 10 | PRISMA | No mention to overlap |
| Nadkarni 2023 | 36134481 | Common strategies in empirically supported psychological interventions for alcohol use disorders: A meta-review. | Narrow | Broad | Broad | 13 | Not reported | In the 'Data extraction / Synthesis and presentation and summary of findings' step: Extracting and analysing primary study data |
| Naghsh 2023 | 36700039 | Profiling Inflammatory Biomarkers following Curcumin Supplementation: An Umbrella Meta-Analysis of Randomized Clinical Trials. | Broad | Narrow | Broad | 10 | PRISMA | Addressed only in the discussion section |
| Nahlén Bose 2023 | 36970265 | A meta-review of systematic reviews and meta-analyses on outcomes of psychosocial interventions in heart failure. | Narrow | Broad | Broad | 7 | Not reported | In the 'Synthesis and presentation and summary of findings' step: Quantifying overlap with a method different than CCA  In the 'Synthesis and presentation and summary of findings' step: Visual representation of overlap through matrices of evidence |
| Nambiar 2023 | 36918855 | Interventions addressing maternal and child health among the urban poor and homeless: an overview of systematic reviews. | Broad | Broad | Broad | 33 | Not reported | No mention to overlap |
| Noeding Fischer 2023 | 36585553 | An appraisal of the methodology and quality of evidence of systematic reviews on the efficacy of prone positional ventilation in adult patients with acute respiratory distress syndrome: an umbrella review. | Narrow | Narrow | Narrow | 16 | PRIOR | In the 'Synthesis and presentation and summary of findings' step: Visual representation of overlap through matrices of evidence  In the 'Synthesis and presentation and summary of findings' step: Select reviews |
| Nowicka 2023 | 36697348 | Metanalyses on metformin's role in pancreatic cancer suffer from severe bias and low data quality - An umbrella review. | Narrow | Narrow | Narrow | 11 | Not reported | In the 'Synthesis and presentation and summary of findings' step: Visual representation of overlap through matrices of evidence  In the 'Data extraction / Synthesis and presentation and summary of findings' step: Extracting and analysing primary study data |
| O'Neill 2023 | 37077169 | Medicinal Cannabis and Implications for Workplace Health and Safety: Scoping Review of Systematic Reviews. | Broad | Narrow | Broad | 31 | PRISMA | No mention to overlap |
| Olaithe 2023 | 36854653 | Sleep in young people: What works now and where to? A meta-review of behavioural and cognitive interventions and lifestyle factors. | Narrow | Broad | Broad | 12 | PRISMA | No mention to overlap |
| Pappas 2023 | 34149334 | The 40-year debate: a meta-review on what works for juvenile offenders. | Narrow | Broad | Broad | 48 | Not reported | In the 'Data extraction / Synthesis and presentation and summary of findings' step: Extracting and analysing primary study data |
| Pathomwichaiwat 2023 | 37471428 | Effects of turmeric (Curcuma longa) supplementation on glucose metabolism in diabetes mellitus and metabolic syndrome: An umbrella review and updated meta-analysis. | Narrow | Narrow | Narrow | 14 | PRISMA | In the 'Synthesis and presentation and summary of findings' step: Quantifying overlap with CCA  In the 'Synthesis and presentation and summary of findings' step: Visual representation of overlap through matrices of evidence |
| Patton 2023 | 35855678 | A meta-review of the impact of compression therapy on venous leg ulcer healing. | Narrow | Narrow | Narrow | 12 | PRISMA | In the 'Data extraction / Synthesis and presentation and summary of findings' step: Extracting and analysing primary study data |
| Petrelli 2023 | 36829806 | Vitamin D3 and COVID-19 Outcomes: An Umbrella Review of Systematic Reviews and Meta-Analyses. | Broad | Narrow | Broad | 27 | MOOSE | No mention to overlap |
| Purssell 2023 | 36798024 | Face mask use to prevent COVID-19 in clinical practice. Using a review of reviews to improve decision-making and transparency. | Broad | Narrow | Broad | 15 | PRIOR | In the 'Data extraction / Synthesis and presentation and summary of findings' step: Extracting and analysing primary study data |
| Qiu 2023 | 37097500 | Promoting physical activity among cancer survivors: an umbrella review of systematic reviews. | Narrow | Broad | Broad | 26 | PRISMA | No mention to overlap |
| Rapisarda 2023 | 37190495 | Outcome Comparison of Drug-Resistant Trigeminal Neuralgia Surgical Treatments-An Umbrella Review of Meta-Analyses and Systematic Reviews. | Narrow | Narrow | Narrow | 10 | PRISMA | No mention to overlap |
| Rapti 2023 | 36981477 | Effects of Exercise and Physical Activity Levels on Childhood Cancer: An Umbrella Review. | Narrow | Broad | Broad | 13 | PRISMA | In the 'Synthesis and presentation and summary of findings' step: Quantifying overlap with a method different than CCA  In the 'Synthesis and presentation and summary of findings' step: Visual representation of overlap through matrices of evidence |
| Rasmussen 2023 | 37080121 | Effectiveness of dementia education for professional care staff and factors influencing staff-related outcomes: An overview of systematic reviews. | Narrow | Broad | Broad | 17 | PRISMA | Addressed only in the discussion section |
| Razak 2023 | 36303465 | Interventions for reducing late-onset sepsis in neonates: an umbrella review. | Narrow | Broad | Broad | 101 | PRIO-harms | No mention to overlap |
| Reis 2023 | 36369785 | Bright light therapy for mental and behavioral illness: A systematic umbrella review. | Broad | Narrow | Broad | 67 | PRISMA | In the 'Synthesis and presentation and summary of findings' step: Select reviews |
| Ribeiro 2023 | 36602588 | Impact of malocclusion treatments on Oral Health-Related Quality of Life: an overview of systematic reviews. | Narrow | Narrow | Narrow | 15 | PRISMA | In the 'Data extraction / Synthesis and presentation and summary of findings' step: Extracting and analysing primary study data |
| Rocha 2023 | 37025068 | Exergaming in the treatment of gait, balance, and quality of life in Parkinson's disease: Overview of systematic reviews. | Narrow | Narrow | Narrow | 9 | PRISMA | In the 'Synthesis and presentation and summary of findings' step: Quantifying overlap with CCA |
| Rodríguez-González 2023 | 36509032 | Effectiveness of interventions using apps to improve physical activity, sedentary behavior and diet: An umbrella review. | Broad | Broad | Broad | 12 | PRISMA | No mention to overlap |
| Sai Krishna 2023 | 36777071 | The Role of Conservative Management in the Avascular Necrosis of the Femoral Head: A Review of Systematic Reviews. | Narrow | Narrow | Narrow | 7 | PRISMA | No mention to overlap |
| Sarmadi 2023 | 37500345 | The effect of cinnamon consumption on lipid profile, oxidative stress, and inflammation biomarkers in adults: An umbrella meta-analysis of randomized controlled trials. | Broad | Narrow | Broad | 11 | PRISMA | Addressed only in the discussion section |
| Sawangjit 2023 | 36728740 | Efficacy and safety of herbal medicine on dementia and cognitive function: An umbrella review of systematic reviews and meta-analysis. | Broad | Broad | Broad | 37 | PRISMA | In the 'eligibility criteria step: Select reviews according to pre-specified eligibility criteria or decision rules |
| Scarpini 2023 | 36629590 | Associated factors and treatment options for sleep bruxism in children: an umbrella review. | Narrow | Broad | Broad | 6 | PRISMA | In the 'Synthesis and presentation and summary of findings' step: Quantifying overlap with CCA  In the 'Synthesis and presentation and summary of findings' step: Visual representation of overlap through matrices of evidence |
| Schuch 2023 | 36963644 | Revisiting the evidence of photodynamic therapy for oral potentially malignant disorders and oral squamous cell carcinoma: An overview of systematic reviews. | Narrow | Narrow | Narrow | 30 | PRISMA | No mention to overlap |
| Semertzidou 2023 | 37072764 | Diabetes and anti-diabetic interventions and the risk of gynaecological and obstetric morbidity: an umbrella review of the literature. | Narrow | Broad | Broad | 21 | PRISMA | In the 'Synthesis and presentation and summary of findings' step: Select reviews  In the 'Synthesis and presentation and summary of findings' step: Statistical methods (e.g., sensitivity analysis) |
| Seow 2023 | 37115231 | Lower re-rupture rates but higher complication rates following surgical versus conservative treatment of acute achilles tendon ruptures: a systematic review of overlapping meta-analyses. | Narrow | Broad | Broad | 34 | PRISMA | In the 'Synthesis and presentation and summary of findings' step: Quantifying overlap with a method different than CCA |
| Sephien 2023 | 36455697 | Efficacy of SGLT2 inhibitors in patients with heart failure: An overview of systematic reviews. | Narrow | Narrow | Narrow | 8 | Not reported | In the 'Synthesis and presentation and summary of findings' step: Quantifying overlap with a method different than CCA |
| Shen 2023 | 37724171 | Transcranial Magnetic Stimulation as a Therapy for Migraine: An Overview of Systematic Reviews. | Narrow | Narrow | Narrow | 7 | Not reported | In the 'Synthesis and presentation and summary of findings' step: Quantifying overlap with CCA  In the 'Synthesis and presentation and summary of findings' step: Visual representation of overlap other than matrices of evidence |
| Shivgulam 2023 | 37017797 | Impact of Exercise Training Interventions on Flow-Mediated Dilation in Adults: An Umbrella Review. | Broad | Broad | Broad | 27 | PRISMA | In the 'Synthesis and presentation and summary of findings' step: Quantifying overlap with a method different than CCA |
| Simon 2023 | 36636142 | Anti-Inflammatory Treatment Efficacy in Major Depressive Disorder: A Systematic Review of Meta-Analyses. | Narrow | Broad | Broad | 20 | PRISMA | In the 'Synthesis and presentation and summary of findings' step: Visual representation of overlap through matrices of evidence |
| Singh 2023 | 36796860 | Effectiveness of physical activity interventions for improving depression, anxiety and distress: an overview of systematic reviews. | Broad | Broad | Broad | 97 | PRISMA | In the 'Synthesis and presentation and summary of findings' step: Quantifying overlap with CCA |
| Só 2023 | 36988825 | Do NSAIDs used prior to standard inferior alveolar nerve blocks improve the analgesia of mandibular molars with irreversible pulpitis? An umbrella review. | Narrow | Narrow | Narrow | 12 | PRISMA | No mention to overlap |
| Solmi 2023 | 35999275 | Efficacy and acceptability of psychosocial interventions in schizophrenia: systematic overview and quality appraisal of the meta-analytic evidence. | Narrow | Broad | Broad | 78 | Not reported | No mention to overlap |
| Talebi 2023 | 37829729 | The effects of pro-, pre-, and synbiotics supplementation on polycystic ovary syndrome: an umbrella review of meta-analyses of randomized controlled trials. | Narrow | Narrow | Narrow | 9 | Not reported | No mention to overlap |
| Talebi 2023 | 36906848 | Early vs delayed enteral nutrition or parenteral nutrition in hospitalized patients: An umbrella review of systematic reviews and meta-analyses of randomized trials. | Narrow | Narrow | Narrow | 45 | PRIOR | In the 'Data extraction' step: Extract a subset of reviews according to prespecified criteria |
| Tan 2023 | 36898857 | Tooth autotransplantation: An umbrella review. | Narrow | Narrow | Narrow | 17 | PRISMA | In the 'Synthesis and presentation and summary of findings' step: Quantifying overlap with CCA  In the 'Synthesis and presentation and summary of findings' step: Visual representation of overlap through matrices of evidence  In the 'Synthesis and presentation and summary of findings' step: Visual representation of overlap other than matrices of evidence |
| Tedla 2023 | 36617689 | Transcranial direct current stimulation (tDCS) effects on upper limb motor function in stroke: an overview review of the systematic reviews. | Narrow | Broad | Broad | 6 | Not reported | No mention to overlap |
| Tonino 2023 | 36875043 | Hemoglobin modulation affects physiology and patient reported outcomes in anemic and non-anemic subjects: An umbrella review. | Narrow | Broad | Broad | 33 | PRISMA | In the 'Synthesis and presentation and summary of findings' step: Select reviews |
| Trembath 2023 | 36081343 | Non-pharmacological interventions for autistic children: An umbrella review. | Narrow | Broad | Broad | 58 | PRISMA | In the 'Synthesis and presentation and summary of findings' step: Quantifying overlap with CCA |
| Tsoi 2023 | 36946438 | The Western and Chinese exercise training for blood pressure reduction among hypertensive patients: An overview of systematic reviews. | Narrow | Broad | Broad | 39 | PRISMA | In the 'Synthesis and presentation and summary of findings' step: Quantifying overlap with CCA  In the 'Synthesis and presentation and summary of findings' step: Visual representation of overlap through matrices of evidence |
| Türk 2023 | 37030086 | Interventions for ADHD in childhood and adolescence: A systematic umbrella review and meta-meta-analysis. | Narrow | Broad | Broad | 16 | PRISMA | In the 'Synthesis and presentation and summary of findings' step: Quantifying overlap with CCA  In the 'Synthesis and presentation and summary of findings' step: Visual representation of overlap through matrices of evidence  In the 'Synthesis and presentation and summary of findings' step: Select reviews |
| Unhapipatpong 2023 | 36898635 | The effect of curcumin supplementation on weight loss and anthropometric indices: an umbrella review and updated meta-analyses of randomized controlled trials. | Broad | Narrow | Broad | 14 | Not reported | In the 'Synthesis and presentation and summary of findings' step: Quantifying overlap with CCA  In the 'Synthesis and presentation and summary of findings' step: Visual representation of overlap through matrices of evidence |
| Verdonschot 2023 | 35947869 | Effectiveness of school-based nutrition intervention components on fruit and vegetable intake and nutrition knowledge in children aged 4-12 years old: an umbrella review. | Narrow | Broad | Broad | 8 | PRISMA | In the 'Synthesis and presentation and summary of findings' step: Quantifying overlap with a method different than CCA  In the 'Synthesis and presentation and summary of findings' step: Visual representation of overlap through matrices of evidence |
| Verma 2023 | 36835915 | An Overview of Systematic Reviews and Meta-Analyses on the Effect of Medication Interventions Targeting Polypharmacy for Frail Older Adults. | Narrow | Broad | Broad | 10 | Not reported | Addressed only in the discussion section |
| Viderman 2023 | 37521355 | Virtual reality for pain management: an umbrella review. | Broad | Narrow | Broad | 21 | PRISMA | In the 'Synthesis and presentation and summary of findings' step: Quantifying overlap with a method different than CCA |
| Vieira 2023 | 36960028 | Telerehabilitation for musculoskeletal pain - An overview of systematic reviews. | Broad | Broad | Broad | 16 | PRISMA | In the 'Synthesis and presentation and summary of findings' step: Quantifying overlap with a method different than CCA  In the 'Synthesis and presentation and summary of findings' step: Visual representation of overlap through matrices of evidence |
| Villar-Alises 2023 | 36674309 | Prenatal Yoga-Based Interventions May Improve Mental Health during Pregnancy: An Overview of Systematic Reviews with Meta-Analysis. | Narrow | Broad | Broad | 10 | PRIO-harms | In the 'Synthesis and presentation and summary of findings' step: Quantifying overlap with CCA  In the 'Synthesis and presentation and summary of findings' step: Visual representation of overlap through matrices of evidence  In the 'Synthesis and presentation and summary of findings' step: Visual representation of overlap other than matrices of evidence |
| Viswanathan 2023 | 37008560 | Is anterior cervical plating necessary for cage constructs in anterior cervical discectomy and fusion surgery for cervical degenerative disorders? Evidence-based on the systematic overview of meta-analyses. | Narrow | Narrow | Narrow | 13 | PRISMA | No mention to overlap |
| Wade 2023 | 36130626 | Utility of healthcare-worker-targeted antimicrobial stewardship interventions in hospitals of low- and lower-middle-income countries: a scoping review of systematic reviews. | Narrow | Broad | Broad | 18 | PRISMA | No mention to overlap |
| Wang 2023 | 37144057 | Is valve-sparing aortic root replacement better than total aortic root replacement? An overview of reviews. | Narrow | Narrow | Narrow | 9 | PRISMA | In the 'eligibility criteria step: Select reviews according to pre-specified eligibility criteria or decision rules |
| Wang 2023 | 37098481 | Chemotherapy and targeted therapy for advanced biliary tract cancers: an umbrella review. | Narrow | Narrow | Narrow | 14 | PRISMA-ScR | In the 'eligibility criteria step: Select reviews according to pre-specified eligibility criteria or decision rules  In the 'Data extraction' step: Extract a subset of reviews according to prespecified criteria |
| Wanjau 2023 | 37790636 | Physical Activity and Depression and Anxiety Disorders: A Systematic Review of Reviews and Assessment of Causality. | Broad | Broad | Broad | 4 | PRISMA | In the 'Synthesis and presentation and summary of findings' step: Quantifying overlap with a method different than CCA |
| Werntz 2023 | 36745497 | Providing Human Support for the Use of Digital Mental Health Interventions: Systematic Meta-review. | Broad | Broad | Broad | 31 | PRISMA | In the 'Synthesis and presentation and summary of findings' step: Quantifying overlap with CCA |
| Whiteley 2023 | 36839218 | Determining Dietary Patterns to Recommend for Type 2 Diabetes: An Umbrella Review. | Narrow | Broad | Broad | 30 | Not reported | Addressed only in the discussion section |
| Witzke 2023 | 36602257 | Specialised nursing tasks in cancer care and their effects. | Broad | Broad | Broad | 11 | PRISMA | In the 'Synthesis and presentation and summary of findings' step: Quantifying overlap with a method different than CCA  In the 'Synthesis and presentation and summary of findings' step: Quantifying overlap with CCA |
| Woldring 2023 | 36662899 | The added value of family-centered rounds in the hospital setting: A systematic review of systematic reviews. | Broad | Narrow | Broad | 4 | PRISMA | In the 'Synthesis and presentation and summary of findings' step: Quantifying overlap with a method different than CCA |
| Wu 2023 | 37074335 | The Effectiveness of Telemedicine in Patients with Rheumatoid Arthritis: An Overview of Systematic Reviews and Meta-Analysis. | Narrow | Broad | Broad | 8 | Not reported | In the 'Data extraction / Synthesis and presentation and summary of findings' step: Extracting and analysing primary study data |
| Wu 2023 | 35971250 | Prevention of unplanned endotracheal extubation in intensive care unit: An overview of systematic reviews. | Narrow | Broad | Broad | 13 | PRIO-harms | No mention to overlap |
| Xu 2023 | 37076415 | Pilates and multiple health outcomes: An umbrella review. | Broad | Broad | Broad | 27 | PRISMA | In the 'Synthesis and presentation and summary of findings' step: Quantifying overlap with CCA  In the 'Synthesis and presentation and summary of findings' step: Visual representation of overlap through matrices of evidence  In the 'Synthesis and presentation and summary of findings' step: Select reviews |
| Yan 2023 | 37817915 | Transarterial Chemoembolization Plus Sorafenib versus Transarterial Chemoembolization Alone for Advanced Hepatocellular Carcinoma: An Umbrella Review of Meta-Analyses and Systematic Reviews. | Narrow | Narrow | Narrow | 12 | PRISMA | No mention to overlap |
| Yang 2023 | 37564180 | Overview of systematic reviews of probiotics in the prevention and treatment of antibiotic-associated diarrhea in children. | Narrow | Narrow | Narrow | 20 | Not reported | Addressed only in the discussion section |
| Yang 2023 | 36635165 | Efficacy and safety of acupuncture for polycystic ovary syndrome: An overview of systematic reviews. | Narrow | Broad | Broad | 11 | PRIO-harms | No mention to overlap |
| Yang 2023 | 36305082 | Effect of pelvic floor muscle training on urinary incontinence after radical prostatectomy: An umbrella review of meta-analysis and systematic review. | Narrow | Narrow | Narrow | 18 | PRISMA | No mention to overlap |
| Ye 2023 | 37814642 | The Effectiveness of Tai Chi for Knee Osteoarthritis: An Overview of Systematic Reviews. | Narrow | Broad | Broad | 6 | PRIOR | No mention to overlap |
| Yi 2023 | 36600678 | Carrot and carotene and multiple health outcomes: an umbrella review of the evidence. | Broad | Narrow | Broad | 30 | Not reported | No mention to overlap |
| Yousafzai 2023 | 36996755 | Clinical efficacy of Azithromycin for COVID-19 management: A systematic meta-analysis of meta-analyses. | Narrow | Narrow | Narrow | 2 | PRISMA | In the 'Synthesis and presentation and summary of findings' step: Quantifying overlap with CCA  In the 'Synthesis and presentation and summary of findings' step: Visual representation of overlap through matrices of evidence |
| Yousuf 2023 | 36601549 | Identification of outcomes reported for hospital antimicrobial stewardship interventions using a systematic review of reviews. | Broad | Broad | Broad | 41 | Not reported | Addressed only in the discussion section |
| Zang 2023 | 35253295 | Effects of different techniques during the second stage of labour on reducing perineal laceration: An overview of systematic reviews. | Narrow | Narrow | Narrow | 18 | PRISMA | No mention to overlap |
| Zarezadeh 2023 | 34817299 | Probiotics act as a potent intervention in improving lipid profile: An umbrella systematic review and meta-analysis. | Narrow | Narrow | Narrow | 38 | Not reported | No mention to overlap |
| Zhang 2023 | 37522003 | Vitamin E intake and multiple health outcomes: an umbrella review. | Broad | Narrow | Broad | 27 | Not reported | No mention to overlap |
| Zhang 2023 | 37256179 | Acupuncture for cancer pain: a scoping review of systematic reviews and meta-analyses. | Narrow | Broad | Broad | 25 | PRISMA-ScR | No mention to overlap |
| Zhang 2023 | 36746815 | Effectiveness of exercise interventions in the management of cancer-related fatigue: a systematic review of systematic reviews. | Narrow | Broad | Broad | 46 | PRISMA | No mention to overlap |
| Zhao 2023 | 37799468 | The effectiveness of exercise on the symptoms in breast cancer patients undergoing adjuvant treatment: an umbrella review of systematic reviews and meta-analyses. | Narrow | Broad | Broad | 15 | Not reported | In the 'Synthesis and presentation and summary of findings' step: Quantifying overlap with CCA |
| Zheng 2023 | 36697778 | Human Albumin Infusion for the Management of Liver Cirrhosis and Its Complications: An Overview of Major Findings from Meta-analyses. | Narrow | Narrow | Narrow | 18 | Not reported | No mention to overlap |
| Zhou 2023 | 37424960 | Scientific Evidence of Acupuncture for Post-Stroke Cognitive Impairment: An Overview of Systematic Reviews and Meta-Analyses. | Narrow | Narrow | Narrow | 15 | Not reported | No mention to overlap |
| Zhou 2023 | 36905961 | Interventions and management on multimorbidity: An overview of systematic reviews. | Narrow | Broad | Broad | 30 | PRISMA | In the 'Synthesis and presentation and summary of findings' step: Quantifying overlap with CCA |
| Zhu 2023 | 37790125 | Association of N-acetylcysteine use with contrast-induced nephropathy: an umbrella review of meta-analyses of randomized clinical trials. | Broad | Narrow | Broad | 12 | PRISMA | In the 'eligibility criteria step: Select reviews according to pre-specified eligibility criteria or decision rules |

^1^A PRISMA diagram alone, without further mention, was insufficient for considering PRISMA as the reporting guideline used.

## Appendix 5. Further details of the overviews selected for the overlap assessment under different scenarios.

| **Study ID** | **PMID** | **Title** | **Population** | **Intervention** | **Overall scope** | **Main outcome** | **Number of SRs** | **Total unique primary references included within the SRs^1^** | **Mean number of primary references per SR** |
| --- | --- | --- | --- | --- | --- | --- | --- | --- | --- |
|  |  |  |  |  |  |  |  |  |  |
| Ambagtsheer | 37088103 | Does CGA Improve Health Outcomes in the Community? An Umbrella Review. | General population of community-dwelling older adults. | Comprehensive geriatric assessment (CGA) delivered in the community to older people (minimum mean age 60 years or where at least 50% of study participants were aged 60 years). | Broad | Not living at home (includes both mortality and nursing home admission). | 11 | 244 | 22.2 |
| Bellon | 36871416 | Effectiveness of orthopaedic treatments on the enlargement of the upper airways: Overview of systematic reviews. | Growing subjects (<16 years)  [from the results section, it is understood that subjects had malocclusion]. | Orthopaedic treatment and/or myofunctional therapy versus no treatment or treatment with different therapeutic means. | Narrow | Enlargement of the upper airway. | 10 | 53 | 5.3 |
| Contillo | 37105550 | Exercise and Protein Supplementation Recommendations for Older Adults With Sarcopenic Obesity: A Meta-Review. | ≥65 years old with a diagnosis of sarcopenic obesity. | Exercise interventions with and without protein supplementation.  Exercise interventions included aerobic, resistance, and/or combined training.  Protein supplementation protocols were selected if the intervention provided intact proteins or amino acids | Broad | Body weight. | 5 | 26 | 5.2 |
| Desaunay | 36853497 | Benefits and Risks of Antidepressant Drugs During Pregnancy: A Systematic Review of Meta-analyses. | Pregnant women | Antidepressant drugs | Narrow | Depression relapse. | 51 | 331 | 6.4 |
| Motrico | 36958130 | Effectiveness of interventions to prevent perinatal depression: An umbrella review of systematic reviews and meta-analysis. | Women during the perinatal period (from pregnancy up to a maximum of one year postpartum) with or without the risk of developing depression | No restriction for the selection of preventive in­terventions was applied. Psychological, educational, psychosocial, pharmacological, physical, lifestyle interventions and alternative therapies among other in­terventions were included.  Control groups allowed were care as usual, no treat­ment, waiting list, attention control or any type of placebo. | Broad | Prevention of perinatal depression. | 19 | 247 | 13.0 |
| Sai Krishna | 36777071 | The Role of Conservative Management in the Avascular Necrosis of the Femoral Head: A Review of Systematic Reviews. | Patients with avascular necrosis of the femoral head due to any pathology including idiopathic and secondary | Conservative therapies like bisphosphonates, hyperbaric oxygen therapy, shock wave therapies such as extracorporeal shock wave therapy, or electrical therapy such as pulsed electromagnetic field. | Narrow | Failures or disease progression. | 7 | 46 | 6.6 |
| Tan | 36898857 | Tooth autotransplantation: An umbrella review. | Patients receiving tooth autotransplantation | Tooth autotransplantation of teeth. Comparison within tooth autotransplantation with respect to treatment protocols used, including (but not limited to) any pre-, peri- or post-operative factors that could affect the prognosis or outcome of tooth autotransplantation, and against other treatment options, including fixed and removable prosthodontics, implants, orthodontic treatment, and no treatment. | Narrow | Success and survival rates. | 16**^2^** | 183 | 11.4 |

^1^Considering the references for scenarios not adjusted for publication-thread (i.e., each reference counting as one, even if related to a unique study).

^2^Tan included 17 reviews, but one was a master thesis from a Lithuanian university for which we did not have access to. Therefore, it was not considered for data extraction purposes

## Appendix 6. Detailed overall and pairwise CCA values for each overview, according to predefined scenarios.

| **Scenario** | **Ambagtsheer** | | **Bellon** | | **Contillo** | | **Desaunay** | | **Motrico** | | **Sai Krishna** | | **Tan** | |
| --- | --- | --- | --- | --- | --- | --- | --- | --- | --- | --- | --- | --- | --- | --- |
|  | **Overall CCA** | **Median pairwise CCA [Q1-Q3]** | **Overall CCA** | **Median pairwise CCA [Q1-Q3]** | **Overall CCA** | **Median pairwise CCA [Q1-Q3]** | **Overall CCA** | **Median pairwise CCA [Q1-Q3]** | **Overall CCA** | **Median pairwise CCA [Q1-Q3]** | **Overall CCA** | **Median pairwise CCA [Q1-Q3]** | **Overall CCA** | **Median pairwise CCA [Q1-Q3]** |
| **1** | 5.2% | 3.7% [0.0%-13.8%] | 7.6% | 0.0% [0.0%-6.3%] | 22.1% | 6.5% [5.1%-23.5%] | 3.4% | 0.0% [0.0%-4.9%] | 1.5% | 0.0% [0.0%-0.0%] | 6.5% | 0.0% [0.0%-3.8%] | 5.2% | 3.2% [0.0%-7.6] |
| **2** | 11.6% | 5.1% [0.0%-19.8%] | 7.6% | 0.0% [0.0%-6.3%] | 22.1% | 6.5% [5.1%-23.5%] | 3.9% | 0.0% [0.0%-5.6%] | 1.7% | 0.0% [0.0%-0.0%] | 6.5% | 0.0% [0.0%-3.8%] | 5.2% | 3.2% [0.0%-7.6%] |
| **3** | 5.8% | 4.2% [0.0%-14.2%] | 7.3% | 0.0% [0.0%-6.7%] | 20.4% | 7.4% [1.4%-21.9%] | 3.3% | 0.0% [0.0%-5.0%] | 1.7% | 0.0% [0.0%-0.0%] | 6.4% | 0.0% [0.0%-4.0%] | 5.0% | 3.4% [0.0%-7.7%] |
| **4** | 8.5% | 4.4% [0.0%-21.0%] | 9.8% | 0.0% [0.0%-7.7%] | 30.7% | 17.9% [6.2%-32.3%] | 4.1% | 0.0% [0.0%-5.9%] | 1.8% | 0.0% [0.0%-0.0%] | 7.6% | 0.0% [0.0%-3.8%] | 5.6% | 3.3% [0.0%-8.3%] |
| **5** | 11.6% | 5.1% [0.0%-19.8%] | 7.3% | 0.0% [0.0%-6.7%] | 20.4% | 7.4% [1.4%-21.9%] | 3.7% | 0.0% [0.0%-5.6%] | 2.0% | 0.0% [0.0%-0.0%] | 6.4% | 0.0% [0.0%-4.0%] | 5.0% | 3.4% [0.0%-7.7%] |
| **6** | 17.3% | 7.1% [0.0%-34.0%] | 9.8% | 0.0% [0.0%-7.7%] | 30.7% | 17.9% [6.2%-32.3%] | 4.8% | 0.0% [0.0%-6.3%] | 2.0% | 0.0% [0.0%-0.0%] | 7.6% | 0.0% [0.0%-3.8%] | 5.6% | 3.3% [0.0%-8.3%] |
| **7** | 9.4% | 4.4% [0.0%-22.2%] | 9.6% | 0.0% [0.0%-8.3%] | 28.6% | 16.6% [1.6%-32.1%] | 4.0% | 0.0% [0.0%-5.9%] | 2.1% | 0.0% [0.0%-0.0%] | 7.6% | 0.0% [0.0%-4.0%] | 5.4% | 3.5% [0.0%-8.3%] |
| **8** | 17.2% | 7.1% [0.0%-32.8%] | 9.6% | 0.0% [0.0%-8.3%] | 28.6% | 16.6% [1.6%-32.1%] | 4.7% | 0.0% [0.0%-6.8%] | 2.4% | 0.0% [0.0%-0.0%] | 7.6% | 0.0% [0.0%-4.0%] | 5.4% | 3.5% [0.0%-8.3%] |
| **9** | 11.3% | 9.0% [0.0%-21.8%] | 9.6% | 0.0% [0.0%-24.6%] | 22.1% | 6.5% [5.1%-23.5%] | 3.4% | 0.0% [0.0%-4.9%] | 3.4% | 0.8% [0.0%-3.8%] | 6.5% | 0.0% [0.0%-3.8%] | 5.2% | 3.2% [0.0%-7.8%] |
| **10** | 11.3% | 9.0% [0.0%-21.8%] | 9.6% | 0.0% [0.0%-24.6%] | 28.6% | 18.2% [0.0%-42.4%] | 4.6% | 0.0% [0.0%-9.2%] | 3.8% | 0.9% [0.0%-3.9%] | 6.5% | 0.0% [0.0%-3.8%] | 5.9% | 3.2% [0.0%-8.1%] |
| **11** | 7.2% | 5.9% [0.0%-14.2%] | 9.2% | 0.0% [0.0%-20.2%] | 20.4% | 7.4% [1.4%-21.9%] | 3.2% | 0.0% [0.0%-4.8%] | 4.1% | 1.0% [0.0%-3.9%] | 6.4% | 0.0% [0.0%-4.0%] | 5.0% | 3.4% [0.0%-7.7%] |
| **12** | 18.7% | 14.2% [0.0%-35.2%] | 13.0% | 0.0% [0.0%-22.8%] | 30.7% | 17.9% [6.2%-32.3%] | 4.1% | 0.0% [0.0%-5.9%] | 4.1% | 0.9% [0.0%-4.1%] | 7.6% | 0.0% [0.0%-3.8%] | 5.6% | 3.3% [0.0%-8.3%] |
| **13** | 11.3% | 9.0% [0.0%-21.8%] | 9.2% | 0.0% [0.0%-20.2%] | 28.2% | 20.0% [0.0%-47.5%] | 4.7% | 0.0% [0.0%-10.0%] | 4.7% | 1.1% [0.0%-3.9%] | 6.4% | 0.0% [0.0%-4.0%] | 5.8% | 3.4% [0.0%-8.0%] |
| **14** | 18.7% | 14.2% [0.0%-35.2%] | 13.0% | 0.0% [0.0%-22.8%] | 33.3% | 20.0% [0.0%-43.3%] | 5.5% | 0.0% [0.0%-10.0%] | 4.6% | 1.0% [0.0%-4.2%] | 7.6% | 0.0% [0.0%-3.8%] | 6.4% | 3.7% [0.0%-8.3%] |
| **15** | 11.7% | 9.4% [0.0%-20.7%] | 12.7% | 0.0% [0.0%-23.0%] | 28.6% | 16.6% [1.6%-32.1%] | 3.9% | 0.0% [0.0%-5.6%] | 5.0% | 1.4% [0.0%-4.2%] | 7.6% | 0.0% [0.0%-4.0%] | 5.4% | 3.5% [0.0%-8.3%] |
| **16** | 18.7% | 14.2% [0.0%-35.2%] | 12.7% | 0.0% [0.0%-23.0%] | 33.3% | 22.2% [0.0%-48.6%] | 5.7% | 0.0% [0.0%-10.7%] | 5.7% | 1.5% [0.0%-4.2%] | 7.6% | 0.0% [0.0%-4.0%] | 6.2% | 3.8% [0.0%-8.4%] |
| **Minimum CCA value across scenarios** | 5.2% | 3.7% | 7.3% | 0.0% | 20.4% | 6.5% | 3.2% | 0.0% | 1.5% | 0.0% | 6.4% | 0.0% | 5.0% | 3.2% |
| **Maximum CCA value across scenarios** | 18.7% | 14.2% | 13.0% | 0.0% | 33.3% | 22.2% | 5.7% | 0.0% | 5.7% | 1.5% | 7.6% | 0.0% | 6.4% | 3.8% |
| **Range** | **13.5%** | **10.5%** | **5.7%** | **0.0%** | **12.9%** | **15.7%** | **2.5%** | **0.0%** | **4.2%** | **1.5%** | **1.2%** | **0.0%** | **1.4%** | **0.6%** |

CCA: Corrected covered area. Q1; Quartile 1. Q3: Quartile 3.

Range represents the difference between the maximum and the minimum value among the different scenarios (which represents the potential maximum impact of assuming different scenarios).
